# Does air pollution exposure affect semen quality? Evidence from a systematic review and meta-analysis of 93,996 Chinese men

**DOI:** 10.3389/fpubh.2023.1219340

**Published:** 2023-08-03

**Authors:** Junjie Liu, Yanpeng Dai, Runqing Li, Jiayi Yuan, Quanxian Wang, Linkai Wang

**Affiliations:** ^1^Henan Human Sperm Bank, The Third Affiliated Hospital of Zhengzhou University, Zhengzhou, China; ^2^Department of Clinical Laboratory, The Third Affiliated Hospital of Zhengzhou University, Zhengzhou, China; ^3^The Neonatal Screening Center in Henan Province, The Third Affiliated Hospital of Zhengzhou University, Zhengzhou, China

**Keywords:** semen analysis, air pollution, particulate matter, meta-analysis, systematic review

## Abstract

**Background:**

Air pollution may impair male fertility, but it remains controversial whether air pollution affects semen quality until now.

**Objectives:**

We undertake a meta-analysis to explore potential impacts of six pollutants exposure during the entire window (0–90 days prior to ejaculation) and critical windows (0–9, 10–14, and 70–90 days prior to ejaculation) on semen quality.

**Methods:**

Seven databases were retrieved for original studies on the effects of six pollutants exposure for 90 days prior to ejaculation on semen quality. The search process does not limit the language and search date. We only included original studies that reported regression coefficients (*β*) with 95% confidence intervals (CIs). The *β* and 95% CIs were pooled using the DerSimonian-Laird random effect models.

**Results:**

PM_2.5_ exposure was related with decreased total sperm number (10–14 lag days) and total motility (10–14, 70–90, and 0–90 lag days). PM_10_ exposure was related with reduced total sperm number (70–90 and 0–90 lag days) and total motility (0–90 lag days). NO_2_ exposure was related with reduced total sperm number (70–90 and 0–90 lag days). SO_2_ exposure was related with declined total motility (0–9, 10–14, 0–90 lag days) and total sperm number (0–90 lag days).

**Conclusion:**

Air pollution affects semen quality making it necessary to limit exposure to air pollution for Chinese men. When implementing protective measures, it is necessary to consider the key period of sperm development.

## Introduction

8–12% of reproductive-age couples are infertile in the world and its prevalence may be increasing (1). Male factors cause 40–50% of infertile couples (2). Total sperm number, sperm concentration, progressive and total motility are commonly adopted to evaluate male reproductive potential. Sperm quality of sperm donors in China’s Henan Province showed a decreasing trend from 2009 to b2019 (3). Although the exact cause remains unclear, air pollution might be a hazard factor for declining semen quality (4).

Particulate matter (PM) pollution included PM ≤ 10 μm (PM_10_) as well as PM ≤2.5 μm (PM_2.5_). Gaseous pollutants included sulfur dioxide (SO_2_), carbon monoxide (CO), nitrogen dioxide (NO_2_), and ozone (O_3_). Due to different economic growth levels and economic development patterns, air pollution varies greatly from place to place (5–9). Air pollution was serious in China due to rapid industrialization (10–13). Air pollution could cause respiratory symptoms (14–16), cardiovascular disease (17–20), kidney disease (21–23), adverse prenatal outcomes (24), and impaired neurodevelopment (25, 26). It remains controversial whether air pollution exposure during the whole sperm development window has an influence on sperm quality (27–47). A meta-analysis of relevant research data is needed.

The growth period of mature sperm is approximately 90 days, including three critical windows: 0–9 days prior to ejaculation (epididymal storage), 10–14 days prior to ejaculation (development of sperm motility), and 70–90 days prior to ejaculation (spermatogenesis) (48). There are fewer studies on which stage of sperm development is most vulnerable to air pollution, but the findings remain controversial (27, 29, 33, 34, 36–40, 42–44, 47). A meta-analysis of relevant research data is needed.

Although there are five systematic review and meta-analyses on whether semen quality is affected by air contaminants (49–53), the measured indicators of the four systematic review and meta-analyses were the mean differences and the exposure periods were not 90 days (49–52). The four systematic review and meta-analyses compare semen quality between men exposed to high levels of air pollution and men exposed to low levels of air pollution and were not standardized when merging the effects of air pollution from different studies (49–52). The main distinction between the reported four meta-analyses and the present work is that we have studied the association air pollution exposure during the whole 90 day period as well as the three critical windows of sperm development. A systematic review and meta-analysis by Xu et al. reported the effect of air pollution exposure during lag 0–90 days or 0–12 weeks on semen quality based on exposure-response relationships but did not report the effect of air pollution exposure during the three critical windows of sperm development (53). The included articles did not include those published in Chinese and those published recently in 2023, and subgroup or sensitivity analyses were also not performed (53). There is still no systematic review on whether semen quality is affected by air pollution exposure during the three critical windows of sperm development.

Therefore, the first meta-analysis was done for analyzing the relation of air pollution exposure during the whole and three critical windows of sperm development and sperm quality in China.

## Methods

The present meta-analysis was performed in compliance with the Preferred Reporting Items for Systematic Reviews and Meta-Analyses (PRISMA) guidelines (54) as well as PRISMA 2020 checklist had been provided in Supplementary Materials A. This meta-analysis was registered on the PROSPERO website (No. CRD42022374712). Literature search.

We retrieved the Cochrane Library, EMBASE, Web of Science, PubMed, VIP, China National Knowledge Infrastructure (CNKI) as well as Wanfang databases for articles. The search process does not limit the language and search date. Only epidemiological observational studies published in Chinese or English would be included. The applied search words and detailed search strategies are shown in Supplementary Table S1; Supplementary Materials B, respectively. Searches were performed independently by RL and JY Disagreement was resolved by a third author (JL)

### Outcomes

Outcomes included total sperm number, sperm concentration, total and progressive motility.

### Inclusion and exclusion criteria

Inclusion criteria were: (a) reporting the effect of at least one air pollutant exposure during the whole window and/or critical stages of sperm development on sperm quality; (b) cross-sectional or cohort studies; (c) reporting regression coefficients (*β*) and 95% confidence intervals (CIs); (d) Chinese males; and (e) English and Chinese articles. The measured indicators of case-control studies were the means and standard deviations (SDs) rather than *β* and 95% CIs.

The following exclusion criteria were adopted: (a) animal studies, case reports, commentaries, reviews, protocols, editorials, conference abstracts, letters, or book chapters; (b) case-control studies; (c) studies in countries other than China; (d) reported shorter or longer exposure period; (e) focused on indoor air pollution; and (f) multivariate logistic regression.

### Study selection

Two authors (RL and JY) conducted the literature selection independently. If any disagreement arose during the selection process, it would be resolved by discussing with the third author (JL).

### Data extraction

Using a standardized form, the following information was extracted independently from eligible publications by two authors (RL and JY): publication year, first author, design of study, region, setting, research period, study subjects, size of the sample, pollutants exposure measurement, outcome, exposure period, statistical model, adjusted confounding factors, adjusted *β* with their corresponding 95% CIs. Through discussion with the third author (JL), any disagreement in the data extraction was resolved. The missing information of the original study was requested by contacting the corresponding author.

### Quality assessment

Quality assessments of eligible publications were executed independently by two researchers (QW and LW). If there was any inconsistent opinion, it would be resolved by discussing with the third researcher (YD). The Newcastle-Ottawa Scale (NOS) checklist was adopted for evaluating the quality of retrospective as well as prospective cohort studies (55). The Joanna Briggs Institute (JBI) critical appraisal checklist was adopted for evaluating the quality of cross-sectional studies (56). Based on the Grading of Recommendations Assessment, Development and Evaluation (GRADE) guidelines (57), the certainty of evidence was started with moderate and further downgraded based on the following items: publication bias, directness, study limitations, consistency, and precision (58, 59), and upgraded for dose-response gradient, strong effect size as well as plausible confounding effect (60).

### Data analyses

If the articles did not give interquartile range (IQR) values or original incremental units of pollutant exposure, we would contact the authors by email. For parts per billion (ppb) units, the following equations were used to convert to μg/m^3^: 1 ppb = 48/22.4 μg/m^3^ (O_3_); 1 ppb = 46/22.4 μg/m^3^ (NO_2_). It was assumed that the standard ratio of 24 h average, 8 h max, and 1 h max was 8:15:20, which was widely used for O_3_ conversion (61–63). To improve comparability, we converted all estimates to 24-h average. The standardized increment was 10 μg/m^3^ in this study, otherwise it would be converted using the following formula (64, 65):

$$\beta_{\left(\mathrm{standardized}\right)}=\beta_{\left(\mathrm{original}\right)}\times \mathrm{Increment}\;\left(10\right)/\mathrm{Increment}\;\left(\mathrm{original}\right)$$

Statistical analyses were conducted with Stata v12.1 (Stata Corp., United States). The β and 95% CIs were combined using the DerSimonian-Laird random effect models. Chi-squared test and *I*^2^ statistics were used to quantify the heterogeneity. Heterogeneity existed when *p* < 0.05 or *I*^2^ > 50% (66). In order to find sources of heterogeneity, we conducted sub-group analyses based on design of the study (cross-sectional and cohort), location (northern and southern China), and exposure assessment approaches (estimating models or monitoring station). Egger’s test as well as funnel plots were adopted for assessing publication bias. Stability of the findings was judged with the help of sensitivity analysis. *p* < 0.05 was statistical significance.

## Results

### Study characteristics

**Figure 1:**
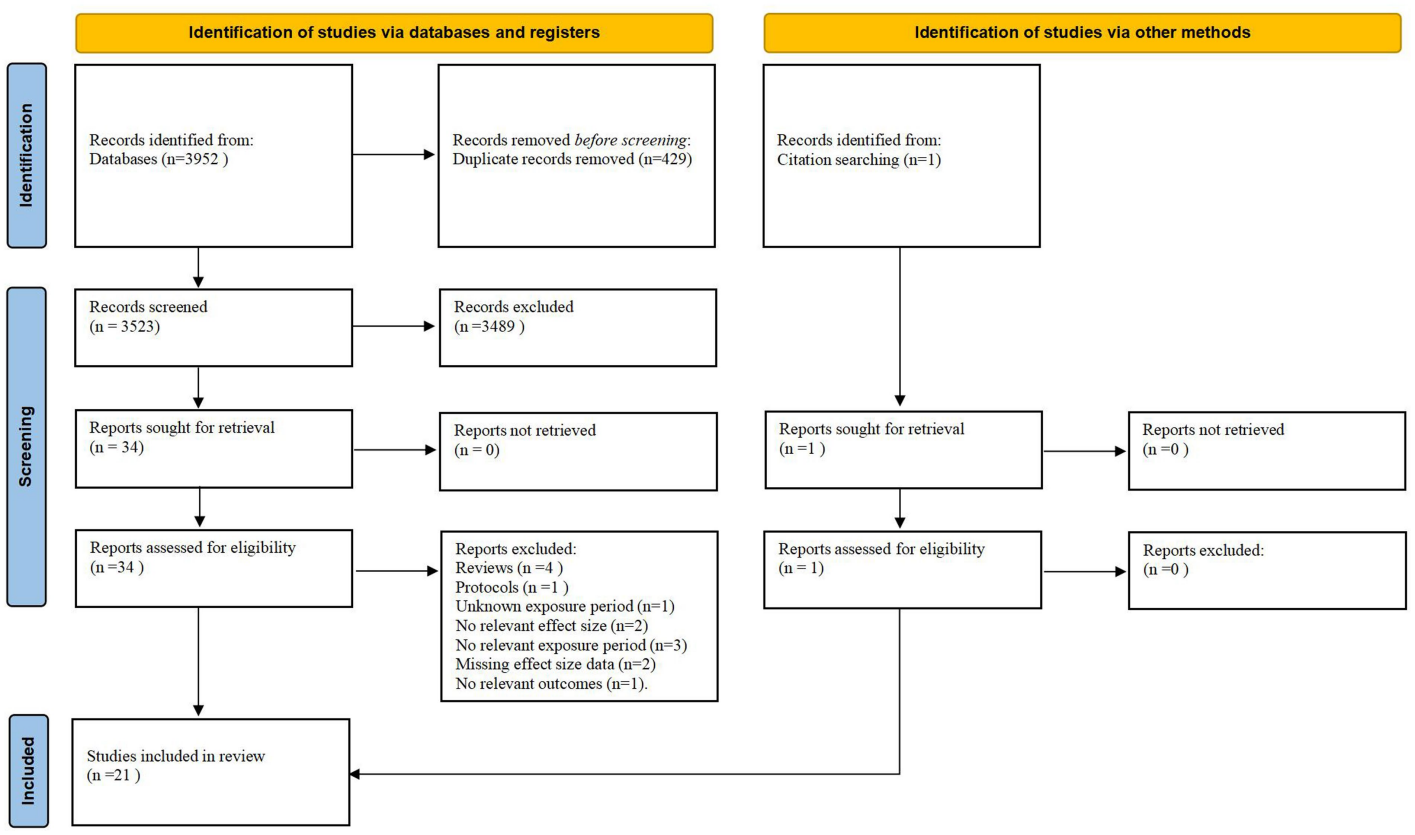
Flow diagram of literature selection.

As depicted in Figure 1, 3,952 publications were retrieved from the seven databases, and 34 articles remained after duplicate literature, abstracts and titles exclusion. After reading the full article, 14 articles were further excluded and detailed exclusion reasons were given in Supplementary Table S2. The remaining 21 eligible publications were eventually included in this meta-analysis. Missing data of original articles were requested by contacting the authors *via* email or WeChat. Studies with missing information were excluded if multiple contacts with the corresponding author remained unanswered. Table 1 illustrates the primary characteristics of the eligible publications. Table 2 demonstrates the original incremental units, outcomes, statistical models used and adjusted confounding factors of all the eligible studies. If the increment unit of the original study was not 10 μg/m^3^, effect sizes were converted. The credibility of the evidence was categorized as very low or low (Supplementary Table S3).

**Table 1:** Characteristics of the included studies in this meta-analysis.

Author publication year	Study design	Location	Setting	Study period	Subjects	Sample size	Exposure measurement	Quality^a^^,^^b^
Dai et al. (2022)	Cohort	East China	Urban	2014–2019	Male partners of infertile couples	1,494 men	Air monitoring station	6 (NOS)
Guan et al. (2020)	Cohort	East China	Urban and rural	2015–2017	Male partners of infertile couples	1955 men with 2073 semen samples	Inverse distance weighting model	7 (NOS)
Huang et al. (2019)	Cohort	Central China	Urban	2014–2015	Male partners of infertile Couples	1,081 with 1,278 semen samples	Air monitoring station	7 (NOS)
Huang et al. (2020)	Cohort	South China	Urban	2018–2019	Sperm donors	1,168 men with 3,797 semen samples	Inverse distance weighting model	8 (NOS)
Lao et al. (2018)	Cross-sectional	East China	Urban and rural	2001–2014	Men from the general population	6,475 men	Hybrid spatiotemporal model	8 (JBI)
Liu et al. (2017)	Cohort	Central China	Urban	2013–2015	Male partners of infertile couples	1759 men with 2,184 semen samples	Inverse distance weighting model	8 (NOS)
Ma et al. (2022)	Cohort	Central China	Urban and rural	2015–2020	Male partners of infertile couples	15,112 men with 28,267 semen samples	Inverse distance weighting model	8 (NOS)
Ma et al. (2023)	Cohort	Central China	Urban and rural	2015–2020	Male partners of infertile couples	15,112 men with 28,267 semen samples	Inverse distance weighting model	8 (NOS)
Qiu et al. (2020)	Cohort	Southwest China	Urban	2013–2018	Sperm donors	686 men with 4,841 semen samples	Air monitoring station	7 (NOS)
Tian et al. (2017)	Cohort	Central China	Urban	2013–2015	Male partners of infertile couples	1780 men	Air monitoring station	7 (NOS)
Wang et al. (2018)	Cross-sectional	Central China	Urban	2013–2015	Male partners of infertile couples	1827 men	Air monitoring station	7 (JBI)
Wang et al. (2020)	Cross-sectional	Central China	Urban and rural	2013–2015	Male partners of infertile couples	1852 men	Air monitoring station	7 (JBI)
Wu et al. (2017)	Cohort	Central China	Urban	2013–2015	Male partners of infertile couples	1759 men with 2,184 semen samples	Inverse distance weighting model	8 (NOS)
Wu et al. (2022)	Cohort	East China	Urban	2014–2016	Fertile men from NUM-LIFE study	1,554 men	Inverse distance weighting model	8 (NOS)
Yu et al. (2022)	Cohort	South China	Urban	2019	Sperm donors	1,310 men with 4,912 semen samples	Land-use random forest model	8 (NOS)
Zhang et al. (2019)	Cohort	North China	Urban	2015–2018	Sperm donors	1,116 men with 8,945 semen samples	Air monitoring station	7 (NOS)
Zhang et al. (2023)	Cohort	East China	Urban	2019–2021	Sperm donors	1,515 men	Inverse distance weighting model	8 (NOS)
Zhao et al. (2022)	Cohort	East China	Urban and rural	2013–2019	Male partners of infertile couples	33,876 men	Air monitoring station	7 (NOS)
Zhou et al. (2014)	Cohort	Southwest China	Urban and rural	2007–2013	Healthy fertile men	1,346 men	Air monitoring station	8 (NOS)
Zhou et al. (2018)	Cohort	Southwest China	Urban	2014–2015	General college students	796 men	Air monitoring station	7 (NOS)
Zhou et al. (2021)	Cross-sectional	North China	Urban and rural	2018–2019	Male partners of infertile couples	423 men	Ordinary Kringing model	8 (JBI)

a The Newcastle-Ottawa Scale (NOS) checklist was adopted for evaluating the quality of retrospective as well as prospective cohort studies and the maximum score is 9.

b The Joanna Briggs Institute (JBI) critical appraisal checklist was adopted for evaluating the quality of cross-sectional studies and the maximum score is 8.

NUM-LIFE, Nanjing Medical University Longitudinal Investigation of Fertility and the Environment; NOS, Newcastle-Ottawa Scale; JBI, Joanna Briggs Institute.

**Table 2:** Pollutants, outcomes, and statistical information of the 19 included studies.

Author publication year	Outcome	Exposure period (day)	Pollutants (Original incremental unit)	Statistical mode	Adjusted confounding factors
Dai et al. (2022)	Sperm concentration Total sperm count Progressive motility Total motility	90	PM_2.5_ (IQR) PM_10_ (IQR)	Multivariate linear regression models	Age, abstinence days, education level, occupation, average ambient temperature, seasons, and gaseous air pollutants
Guan et al. (2020)	Sperm concentration Total sperm count Progressive motility Total motility	90	PM_2.5_ (IQR) PM_10_ (IQR)	Multivariate linear regression models	Age, abstinence days, semen volume
Huang et al. (2019)	Sperm concentration Total sperm count Total motility	90	PM_2.5_ (IQR)	Multivariate linear mixed models	Age, BMI, race, education, smoking, alcohol consumption, abstinence period, and season
Huang et al. (2020)	Sperm concentration Total sperm count Progressive motility Total motility	90	PM_2.5_ (μg/m^3^) PM_10_ (μg/m^3^) SO_2_ (μg/m^3^) NO_2_ (μg/m^3^) CO (mg/m^3^)	Linear mixed-effect models	Age, BMI, percent body fat, ethnic, marital status, childbearing history, career, smoking, alcohol consumption, abstinence period, season, a natural cubic spline function of time, a natural cubic spline function of temperature during exposure period
Lao et al. (2018)	Sperm concentration Progressive motility Total motility Percentage of normal morphology	90	PM_2.5_ (5 μg/m^3^)	Multivariate linear regression models	Age, education level, smoking status, alcohol drinking, exercise and occupational exposure to asbestos and organic solvent, body mass index, systolic blood pressure, fasting blood glucose and total cholesterol levels, season, year of medical examination
Liu et al. (2017)	Sperm concentration Total sperm count Progressive motility Total motility Total motile sperm count	90	SO_2_ (IQR) NO_2_ (IQR) CO (IQR) O_3_ (IQR)	Multiple linear regression analysis	Age, BMI, race, education, smoking amount, alcohol consumption, and abstinence period, temperature, season
Ma et al. (2022)	Sperm concentration Total sperm count Progressive motility Total motility Progressively motile sperm count Total motile sperm count	90	PM_2.5_ (IQR) PM_10_ (IQR)	Linear mixed-effects models	Age, BMI, smoking, drinking, occupation, abstinence period, month (at the date of semen collection) and temperature (average temperature of contemporary period)
Ma et al. (2023)	Sperm concentration Total sperm count Progressive motility Total motility Progressively motile sperm count Total motile sperm count	90	SO_2_ (IQR) NO_2_ (IQR) CO (IQR) O_3_ (IQR)	Linear mixed-effects models	Age, BMI, smoking, drinking, occupation, abstinence period, month (at the date of semen collection) and temperature (average temperature of contemporary period)
Qiu et al. (2020)	Semen volume Sperm concentration Progressive motility	90	PM_2.5_ (μg/m^3^) PM_10_ (μg/m^3^) SO_2_ (μg/m^3^) NO_2_ (μg/m^3^) CO (mg/m^3^) O_3_ (1ppb)	Linear mixed-effects models	Abstinence days, age, BMI, education level, year of sample collection, relative humidity (current day, 90-day preceding), temperature (current day, 90-day preceding)
Tian et al. (2017)	Sperm concentration Total sperm count	90	O_3_ (μg/m^3^)	Multivariate linear mixed models	Age, BMI, education level, smoking status, seasons of semen collection, abstinence days, average temperature, average relative humidity
Wang et al. (2018)	Semen volume Sperm concentration Total sperm count Progressive motility Total motility	90	PM_10_ (10 μg/m^3^)	Multiple linear regression analysis	Age, BMI, education level, smoking status, abstinence days, seasons of semen collection, average temperature, average relative humidity
Wang et al. (2020)	Sperm concentration Total sperm count Progressive motility	90	SO_2_ (IQR) NO_2_ (IQR)	Multivariate linear regression models	BMI, education level, smoking, age, and abstinence period, temperature, humidity, season, and PM2.5
Wu et al., (2017)	Sperm concentration Total sperm count Progressive motility Total motility	90	PM_2.5_ (IQR) PM_10_ (IQR)	Multivariate linear regression models	Age, BMI, ethnic, education, smoking, alcohol consumption, abstinence period, season and temperature
Wu et al. (2022)	Semen volume Sperm concentration Total sperm count Progressive motility Total motility	90	PM_2.5_ (10 μg/m^3^)	Multivariate linear regression models	Age, BMI, ethnicity, education, smoking status, drinking status, family income, abstinence period, season, and temperature
Yu et al. (2022)	Sperm concentration Total sperm count Progressive motility Total motility	90	PM_2.5_ (IQR) PM_10_ (IQR)	Linear mixed-effect models	Age. BMI, percent body fat, education, ethnic, martial status, childbearing history, career, smoking, drinking, abstinence period, month, a natural cubic spline function of temperature during exposure period
Zhang et al. (2019)	Sperm concentration Progressive motility	90	PM_2.5_ (μg/m^3^) PM_10_ (μg/m^3^) SO_2_ (μg/m^3^) NO_2_ (μg/m^3^) CO (mg/m^3^) O_3_ (μg/m^3^)	Linear mixed-effect models	Age, abstinence duration, month, average temperature
Zhang et al. (2023)	Sperm concentration Total sperm count Progressive motility Total motility	90	PM_2.5_ (μg/m^3^) PM_10_ (μg/m^3^) SO_2_ (μg/m^3^) NO_2_ (μg/m^3^) CO (μg/m^3^) O_3_ (μg/m^3^)	Multivariate linear regression models	Age, ethnicity, season of semen collection, abstinence period and temperature.
Zhao et al. (2022)	Sperm concentration Total sperm count Progressive motility Total motility	90	PM_2.5_ (IQR) PM_10_ (IQR)	Linear mixed-effect models	Ethnicity, age, educational level, body mass index, smoking, alcohol consumption, season of semen collection, abstinence period, temperature, relative humidity, and gaseous pollutants
Zhou et al. (2014)	Semen volume Sperm concentration Progressive motility Total motility Percentage of normal morphology	90	PM_10_ (μg/m^3^) SO_2_ (μg/m^3^) NO_2_ (μg/m^3^)	Multiple linear regression analysis	Age, education, smoking, BMI, alcohol use, abstinence time period and season
Zhou et al. (2018)	Semen volume Sperm concentration Total sperm count Progressive motility Percentage of normal morphology	90	PM_2.5_ (μg/m^3^) PM_10_ (μg/m^3^)	Multiple linear regression analysis	Age, smoking, alcohol use, BMI and abstinence time
Zhou et al. (2021)	Sperm concentration Total sperm count Progressive motility Total motility	90	PM_2.5_ (μg/m^3^) PM_10_ (μg/m^3^) SO_2_ (μg/m^3^) NO_2_ (μg/m^3^) CO (mg/m^3^) O_3_ (μg/m^3^)	Multiple linear regression models	Abstinence, age, BMI, socioeconomic status, smoking status, alcohol consumption, psychological stress, exposures to heat, metals or solvents, average ambient air temperature, multi-time windows and multi-pollutants

PM_2.5_, particulate matter with the diameter ≤ 2.5 μm; PM_10_, particulate matter with diameter ≤ 10 μm; SO_2_, sulfur dioxide; NO_2_, nitrogen dioxide; CO, carbon monoxide; O_3_, ozone; BMI, body mass index; IQR, inter-quartile rages (IQR).

### Air pollutants and sperm quality

**Figure 2:**
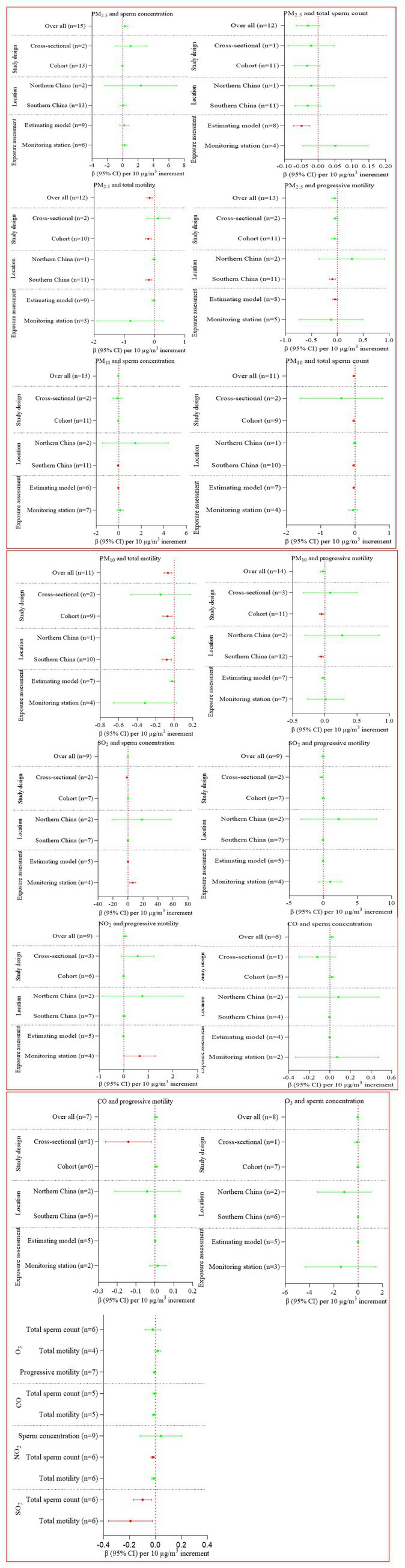
Regression coefficients and 95% confidence intervals for the relation between six pollutants exposure during the whole window and sperm quality.

Six air pollutants exposure during the whole window did not affect sperm concentration (Supplementary Table S4; Figure 2). PM_10_, SO_2_, and NO_2_ exposure during the whole window were related with decreased total sperm number, while such association was not found for PM_2.5_, CO, and O_3_ exposure (Supplementary Table S4; Figure 2). PM_2.5_, PM_10_ as well as SO_2_ exposure during the entire window were negatively related with total motility, while such association was not found for other pollutants.

**Figure 3:**
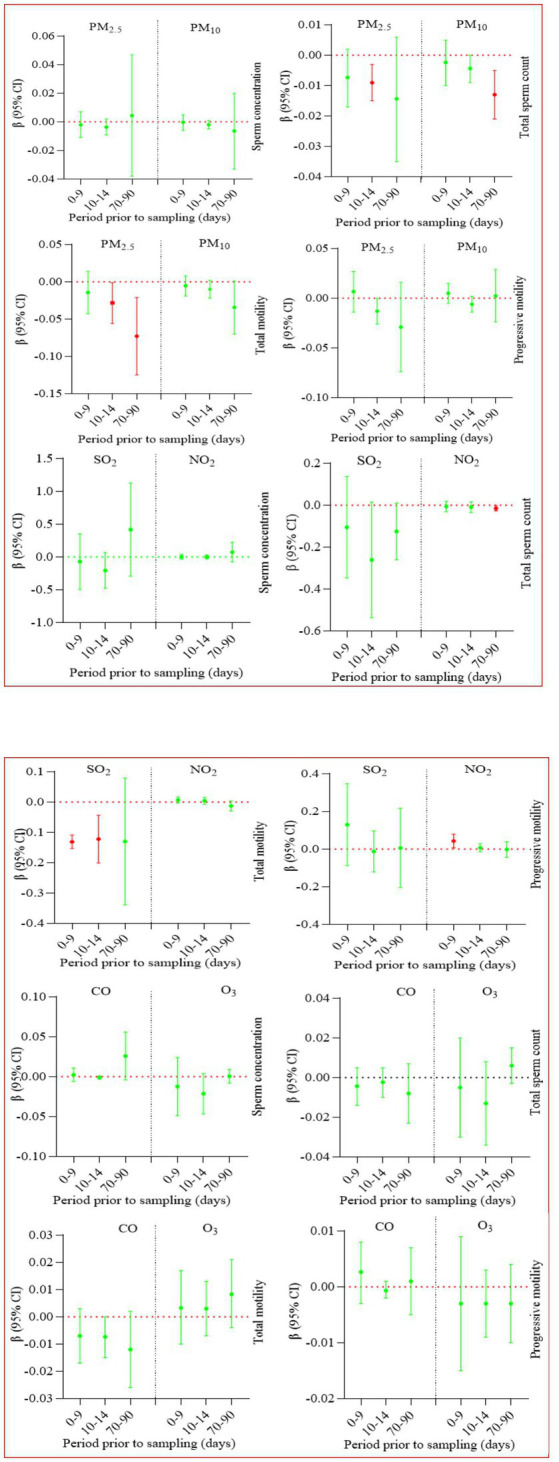
Regression coefficients and 95% confidence intervals for the relation between six pollutants exposure during three critical windows and sperm quality.

In order to find sources of heterogeneity, we conducted sub-group analyses based on design of the study (cohort and cross-sectional), location (northern China and southern China), and exposure assessment approaches (monitoring station or estimating models). The majority of sub-group results were consistent with the pooled results (Supplementary Table S5; Figure 3).

During 0–9 lag days, only SO_2_ exposure was related with declined total motility (Supplementary Table S5; Figure 3). During 10–14 lag days, PM_.2.5_ exposure was adversely related with total sperm number and total motility, SO_2_ with total motility (Supplementary Table S5; Figure 3). During 70–90 lag days, PM_10_ and NO_2_ exposure were adversely related with total sperm number, PM_2.5_ with total motility (Supplementary Table S5; Figure 3).

### Sensitivity analysis

In the sensitivity analyses for six pollutants exposure during the whole window and sperm quality, pooled effect sizes did not change significantly by omitting one study from each analysis, thus indicating that our findings were stable (Supplementary Table S4; Supplementary Figure S1). However, when the study of Wu et al. (40) was omitted from sensitivity analyses of PM_2.5_ exposure and progressive motility, a significant association disappeared (*p* = 0.081; Supplementary Table S4; Supplementary Figure S1A). When the study of (34) was omitted from sensitivity analyses of PM_10_ exposure and sperm concentration, a significant association disappeared (*p* = 0.119; Supplementary Table S4; Supplementary Figure S1B). When the study by Ma et al. (33) was omitted from the sensitivity analysis of O_3_ exposure and total motility, a significant association disappeared (*p* = 0.104; Supplementary Table S4; Supplementary Figure S1F).

In the sensitivity analyses of six pollutants exposure during critical windows and sperm quality, the pooled effect sizes did not change significantly by omitting one study from each analysis, thus indicating that our findings were stable. However, when the study of Ma et al. (33) was omitted from the sensitivity analyses of O_3_ (70–90 lag days) exposure and total motility, a significant association disappeared (*p* = 0.197) with heterogeneity decreasing from 51 to 0% (Supplementary Table S5).

## Discussion

### Summary of study results

**Figure 4:**
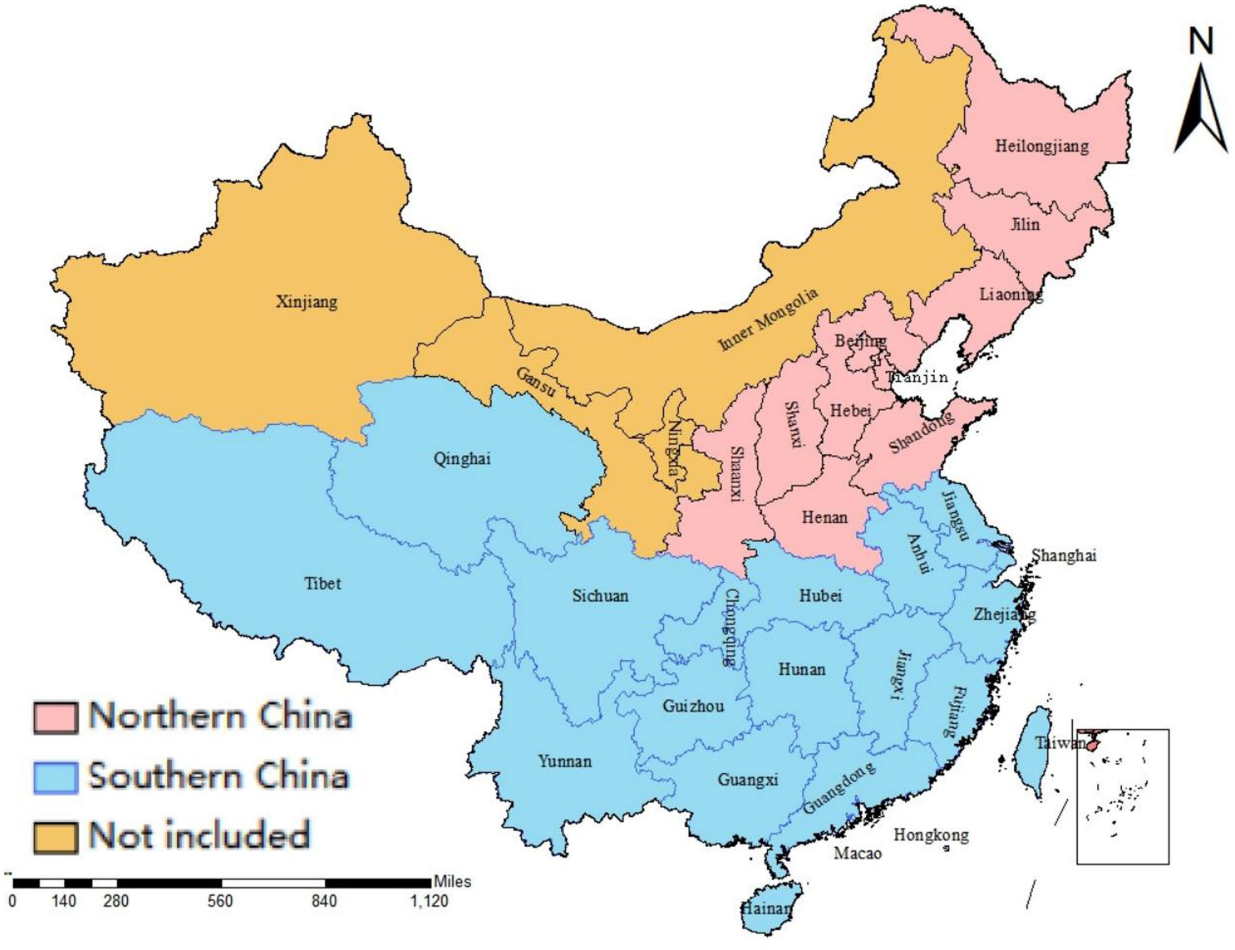
Distribution of northern and southern China.

China has a population of more than 1.4 billion and covers a land area of approximately 9.6 million km^2^. Due to the vast territory of China, it varies greatly in climate conditions, landforms, geography, population density, and economic development level in different regions. Based on economic development levels and climatic conditions, China is generally grouped into seven geographic regions (67–69). Detailed geographic location is presented in Supplementary Figure S2. China is roughly classified as southern and northern China (70–72). Distribution of southern and northern China is shown in Figure 4. As a result of the limited sample size, we performed sub-group analysis by location (northern China and southern China). Air quality is closely related with climatic conditions and economic development levels. Air quality is better in western China than in eastern China (67). Economic development levels in western and eastern regions result in different chemical compositions of pollutants (73, 74). In the eastern and central regions, industry and traffic are the primary causes of air pollution (75). Biomass burning and soil dust are the primary reasons of air pollution in the western region. Different sources of air pollution in different regions result in different toxicity, concentrations, and chemical compositions. This may explain, to some extent, the inconsistent results.

Different individual exposure assessment approaches can partially explain the controversial results. Lao et al. estimated individual exposure levels of PM_2.5_ using a high-resolution (1 × 1 km) spatiotemporal model (31). Zhou et al. (44) adopted the ordinary Kriging model to measure individual exposure concentrations. Some studies used the land-use random forest model (41) or inverse distance weighting model (28, 29, 32, 34, 39, 40) to assess the actual individual exposure levels. Some other studies used the averaged levels of the city-wide or the nearest monitoring station to assess actual individual pollutant exposure concentrations (27, 30, 35–38, 42, 43, 45, 46).

This is the first meta-analysis to analyze potential impacts of ambient air pollution exposure during the whole window and three critical windows on semen quality in China. Sperm motility, a conventional semen parameter, is one of the common indicators of fertility assessment. Sperm motility is commonly used as one of the most important sperm functions to determine whether female partners can successfully conceive without any assisted reproductive technology (ART). Sperm motility parameters are also sensitive indicators of male reproductive toxicity (76). PM_10_, PM_2.5_ as well as SO_2_ exposure were adversely related with total motility during 0–90 days prior to ejaculation. PM_2.5_, CO as well as O_3_ exposure were adversely related with total sperm number during 0–90 lag days.

In order to find sources of heterogeneity, we conducted sub-group analyses based on design of the study (cohort and cross-sectional), location (northern China and southern China), and exposure assessment approaches (monitoring station or estimating models). Although subgroup analysis reduced heterogeneity to some extent, heterogeneity remained high level in some subgroups, and it was necessary to continue to explore potential sources of between-studies heterogeneity.

In addition, the possible exposure susceptibility window was also investigated. PM_2.5_ exposure affected total motility (10–14 and 70–90 lag days) and total sperm number (10–14 lag days). PM_10_ affected total sperm number (70–90 lag days). SO_2_ influenced total sperm number (0–9 and 10–14 lag days). NO_2_ affected total sperm number (70–90 lag days). The findings suggested that pollutants exposure might affect total motility and total sperm number.

### Biological mechanisms

The biological mechanisms that environmental pollutant exposure may damage the development of total motility have not been elucidated. PM_10_, PM_2.5_, and O_3_ exposure can lead to elevated concentrations of reactive oxygen species (77, 78), which may disrupt the blood-testis barrier, detriment spermatogenesis and result in declined sperm motility (79–82). PM exposure can also cause systemic inflammatory reactions by elevating tumor necrosis factor (TNF) as well as interleukin-1β (IL-1β) levels (83–86). Higher concentrations of IL-1β and TNF are related with impaired total sperm motility (87–89). Significant reduction in air pollutants emissions was accompanied by improvements in people’s markers of inflammatory conditions, thrombosis as well as oxidation stress (90). We hypothesized that environmental pollutant exposure would elevate oxidative stress levels and inflammatory reactions, which could lead to decreased total sperm motility. This hypothesis requires further toxicological studies to elucidate the detailed mechanism of reduced sperm motility caused by environmental pollutant exposure.

## Strengths and limitations

This present meta-analysis has three advantages. First, it is the first meta-analysis to analyze whether semen quality is affected by air pollution exposure during the whole and critical windows. Second, the findings are relatively new as a result of most eligible studies being published within recent 4 years. Third, results of different original studies were difficult to compare since the exposure increment units were different in most cases. Therefore, the comparability of the results was improved by standardizing the data through transformation.

However, the present meta-analysis still has four limitations. First, a high degree of heterogeneity for some pollutants was found, which may be explained by differences in pollutant concentrations, types of air pollutants, chemical components of particulate matter, individual exposure assessment approaches, design of the study, study setting, sample size, study regions, selection bias, and adjustment confounding factors. Due to the high degree of heterogeneity, caution should be given when interpreting some pooled effects. A high degree of heterogeneity may also hinder the detection of publication bias. Second, selective bias may occur due to some of the included studies selecting patients from infertility clinics. Third, subgroup analysis by exposure assessment approaches was not performed as a result of the insufficient sample size. Fourth, the sample size is still inadequate, with only 2 articles from northern China being included. Insufficient data might lead to inescapable errors, and the original researches need to be further supplemented. Fifth, many of the included studies obtained estimates of air pollution exposure from ecological data or modeling and did not examine individual exposure to air pollution.

## Conclusion

This evidence suggested that ambient air pollution could reduce semen quality in Chinese men and may even lead to infertility. For Chinese men, there is a need to reduce the duration of exposure. Further studies should be conducted to explore the possible biological mechanisms behind the findings observed in this study.

## Author contributions

JL and YD proposed the idea and designed the present study, interpreted the findings, and were responsible for statistical analysis and manuscript writing. RL and JY performed literature retrieval, study selection, and data extraction. QW and LW performed the quality assessment. All authors contributed to the article and approved the submitted version.

## Funding

The present study was funded by the Henan Provincial Science and Technology Research Project (No. LHGJ20190389).

## Conflict of interest

The authors declare that the research was conducted in the absence of any commercial or financial relationships that could be construed as a potential conflict of interest.

## Publisher’s note

All claims expressed in this article are solely those of the authors and do not necessarily represent those of their affiliated organizations, or those of the publisher, the editors and the reviewers. Any product that may be evaluated in this article, or claim that may be made by its manufacturer, is not guaranteed or endorsed by the publisher.
