# Supplementary material for: Does air pollution exposure affect semen quality? Evidence from a systematic review and meta-analysis of 93,996 Chinese men
Source: Front Public Health. 2023 Aug 3;11:1219340. doi: 10.3389/fpubh.2023.1219340 (PMC10435904; doi:10.3389/fpubh.2023.1219340)
Supplement: Supplementary file 1 [file Data_Sheet_1.pdf]

Supplementary Materials for

Does air pollution exposure affect semen quality? Evidence from a systematic review and meta-analysis of 93996 Chinese men

Junjie Liu[a†*], Yanpeng Dai[b†], Runqing Li[c], Jiayi Yuan[c], Quanxian Wang[a], Linkai Wang[a]

[a]*Henan Human Sperm Bank, the Third Affiliated Hospital of Zhengzhou University, Zhengzhou, China*

[b]*Department of Clinical Laboratory, the Third Affiliated Hospital of Zhengzhou University, Zhengzhou, China*

[c]*The Neonatal Screening Center in Henan Province, the Third Affiliated Hospital of Zhengzhou University, Zhengzhou, China*

*Corresponding author: zdsfyljj@zzu.edu.cn (J. Liu).

[†]These authors contributed to equally to this article.

Supplementary Materials for the detailed search strategy for database 1 (PubMed)

Search	Actions	Details	Query	Results	Time
#1	•••		Search: **"Air Pollution"[Mesh]** Sort by: **Most Recent**	67,917	05:04:00
#2	•••		Search: **((("Air Pollution"[Mesh]) OR (Air Pollutions[Title/Abstract])) OR (Pollution, Air[Title/Abstract])) OR (Air Quality[Title/Abstract])**	76,162	05:04:13
#3	•••		Search: **"Air Pollutants"[Mesh]** Sort by: **Most Recent**	74,995	05:04:29
#4	•••		Search: **((((((((("Air Pollutants"[Mesh]) OR (Pollutants, Air[Title/Abstract])) OR (Air Pollutant[Title/Abstract])) OR (Pollutant, Air[Title/Abstract])) OR (Air Pollutants, Environmental[Title/Abstract])) OR (Environmental Pollutants, Air[Title/Abstract])) OR (Air Environmental Pollutants[Title/Abstract])) OR (Pollutants, Air Environmental[Title/Abstract])) OR (Environmental Air Pollutants[Title/Abstract])) OR (Pollutants, Environmental Air[Title/Abstract])**	76,557	05:04:37
#5	•••		Search: **"Sulfur Dioxide"[Mesh]** Sort by: **Most Recent**	5,825	05:04:49
#6	•••		Search: **("Sulfur Dioxide"[Mesh]) OR (Sulfurous Anhydride[Title/Abstract])**	5,828	05:04:56
#7	•••		Search: **"Nitrogen Dioxide"[Mesh]** Sort by: **Most Recent**	6,115	05:05:07
#8	•••		Search: **((("Nitrogen Dioxide"[Mesh]) OR (Dioxide, Nitrogen[Title/Abstract])) OR (Nitrogen Peroxide[Title/Abstract])) OR (Peroxide, Nitrogen[Title/Abstract])**	6,383	05:13:38
#9	•••		Search: **"Carbon Monoxide"[Mesh]** Sort by: **Most Recent**	18,941	05:13:48
#10	•••		Search: **("Carbon Monoxide"[Mesh]) OR (Monoxide, Carbon[Title/Abstract])**	19,028	05:14:00
#11	•••		Search: **"Ozone"[Mesh]** Sort by: **Most Recent**	17,110	05:14:10
#12	•••		Search: **((((((("Ozone"[Mesh]) OR (Tropospheric Ozone[Title/Abstract])) OR (Ozone, Tropospheric[Title/Abstract])) OR (Level Ozone, Low[Title/Abstract])) OR (Low Level Ozone[Title/Abstract])) OR (Ozone, Low Level[Title/Abstract])) OR (Ground Level Ozone[Title/Abstract])) OR (Level Ozone, Ground[Title/Abstract])) OR (Ozone, Ground Level[Title/Abstract])**	17,689	05:14:23
#13	•••		Search: **"Particulate Matter"[Mesh]** Sort by: **Most Recent**	75,963	05:14:31
#14	•••		Search: **(((((((((((((Ultrafine Fibers[Title/Abstract]) OR (Ultrafine Fiber[Title/Abstract])) OR (Fiber, Ultrafine[Title/Abstract])) OR (Airborne Particulate Matter[Title/Abstract])) OR (Particulate Matter, Airborne[Title/Abstract])) OR (Air Pollutants, Particulate[Title/Abstract])) OR (Particulate Air Pollutants[Title/Abstract])) OR (Ambient Particulate Matter[Title/Abstract])) OR (Particulate Matter, Ambient[Title/Abstract])) OR (Ultrafine Particulate Matter[Title/Abstract])) OR (Particulate Matter, Ultrafine[Title/Abstract])) OR (Ultrafine Particles[Title/Abstract])) OR (Particles, Ultrafine[Title/Abstract])) OR (Ultrafine Particle[Title/Abstract])) OR (Particle, Ultrafine[Title/Abstract])**	5,397	05:14:38
#15	•••		Search: **"Semen Analysis"[Mesh]** Sort by: **Most Recent**	28,541	05:14:48
#16	•••		Search: **((((((((("Semen Analysis"[Mesh]) OR (Semen Analyses[Title/Abstract])) OR (Semen Quality Analysis[Title/Abstract])) OR (Analyses, Semen Quality[Title/Abstract])) OR (Analysis, Semen Quality[Title/Abstract])) OR (Quality Analyses, Semen[Title/Abstract])) OR (Semen Quality Analyses[Title/Abstract])) OR (Semen Quality[Title/Abstract])) OR (Qualities, Semen[Title/Abstract])) OR (Quality, Semen[Title/Abstract])**	33,163	05:14:57
#17	•••		Search: **"Sperm Count"[Mesh]** Sort by: **Most Recent**	10,947	05:15:03

The detailed search strategy for database1: PubMed (*continued*)

#18	•••	Search: **((((((("Sperm Count"[Mesh]) OR (Count, Sperm[Title/Abstract])) OR (Counts, Sperm[Title/Abstract])) OR (Sperm Counts[Title/Abstract])) OR (Sperm Number[Title/Abstract])) OR (Number, Sperm[Title/Abstract])) OR (Numbers, Sperm[Title/Abstract])) OR (Sperm Numbers[Title/Abstract])**	12,944	05:15:11
#19	•••	Search: **"Sperm Motility"[Mesh]** Sort by: **Most Recent**	19,962	05:15:22
#20	•••	Search: **((("Sperm Motility"[Mesh]) OR (Motilities, Sperm[Title/Abstract])) OR (Motility, Sperm[Title/Abstract])) OR (Sperm Motilities[Title/Abstract])**	20,198	05:15:28
#21	•••	Search: **#2 OR #4 OR #6 OR #8 OR #10 OR #12 OR #14**	161,868	05:15:34
#22	•••	Search: **#16 OR #18 OR #20**	34,671	05:15:40
#23	•••	Search: **#21 AND #22**	182	05:15:49

The detailed search strategy for database 2: Web of Science

☐	1	TS=(Air pollution OR Air Pollutions OR Pollution, Air OR Air Quality)	972,182
☐	2	TS=(Air Pollutants OR Pollutants, Air OR Air Pollutant OR Pollutant, Air OR Air Pollutants, Environmental OR Environmental Pollutants, Air OR Air Environmental Pollutants OR Pollutants, Air Environmental OR Environmental Air Pollutants OR Pollutants, Environmental Air)	495,461
☐	3	TS=(Sulfur dioxide OR Sulfurous Anhydride)	52,857
☐	4	TS=(Nitrogen dioxide OR Dioxide, Nitrogen OR Nitrogen Peroxide OR Peroxide, Nitrogen)	115,306
☐	5	TS=(Carbon monoxide OR Monoxide, Carbon)	165,288
☐	6	TS=(Ozone OR Tropospheric Ozone OR Ozone, Tropospheric OR Level Ozone, Low OR Low Level Ozone OR Ozone, Low Level OR Ground Level Ozone OR Level Ozone, Ground OR Ozone, Ground Level)	125,283
☐	7	OR Airborne Particulate Matter OR Particulate Matter, Airborne OR Air Pollutants, Particulate OR Particulate Air Pollutants OR Ambient Particulate Matter OR Particulate Matter, Ambient OR Ultrafine Particulate Matter OR Particulate Matter, Ultrafine OR Ultrafine Particles OR Particles, Ultrafine OR Ultrafine Particle OR Particle, Ultrafine)	143,418
☐	8	TS=(Semen Analysis OR Semen Analyses OR Semen Quality Analysis OR Analyses, Semen Quality OR Analysis, Semen Quality OR Quality Analyses, Semen OR Semen Quality Analyses OR Semen Quality OR Qualities, Semen OR Quality, Semen OR Semen Qualities)	46,906
☐	9	TS=(Sperm Count OR Count, Sperm OR Counts, Sperm OR Sperm Counts OR Sperm Number OR Number, Sperm OR Numbers, Sperm OR Sperm Numbers)	44,887
☐	10	TS=(Sperm Motility OR Motilities, Sperm OR Motility, Sperm OR Sperm Motilities)	43,621
☐	11	#1 OR #2 OR #3 OR #4 OR #5 OR #6 OR #7	1,378,817
☐	12	#8 OR #9 OR #10	96,475
☐	13	#11 AND #12	1,561

The detailed search strategy for database 3: EMBASE

**#1**	**'air pollution'**/exp	199,515
**#2**	**'air pollutions'**:ab,ti OR **'pollution, air'**:ab,ti OR **'air quality'**:ab,ti	23,090
**#3**	**'air pollutant'**/exp	97,087
**#4**	**'pollutants, air'**:ab,ti OR **'air pollutant'**:ab,ti OR **'pollutant, air'**:ab,ti OR **'air pollutants, environmental'**:ab,ti OR **'environmental pollutants, air'**:ab,ti OR **'air environmental pollutants'**:ab,ti OR **'pollutants, air environmental'**:ab,ti OR **'environmental air pollutants'**:ab,ti OR **'pollutants, environmental air'**:ab,ti	4,792
**#5**	**'sulfur dioxide'**/exp	17,329
**#6**	**'sulfurous anhydride'**:ab,ti	27
**#7**	**'nitrogen dioxide'**/exp	17,060
**#8**	**'dioxide, nitrogen'**:ab,ti OR **'nitrogen peroxide'**:ab,ti OR **'peroxide, nitrogen'**:ab,ti	438
**#9**	**'carbon monoxide'**/exp	44,169
**#10**	**'monoxide, carbon'**:ab,ti	182
**#11**	**'ozone'**/exp	33,310
**#12**	**'tropospheric ozone'**:ab,ti OR **'ozone, tropospheric'**:ab,ti OR **'level ozone, low'**:ab,ti OR **'low level ozone'**:ab,ti OR **'ozone, low level'**:ab,ti OR **'ground level ozone'**:ab,ti OR **'level ozone, ground'**:ab,ti OR **'ozone, ground level'**:ab,ti	2,319
**#13**	**'particulate matter'**/exp	57,202
**#14**	**'ultrafine fibers'**:ab,ti OR **'ultrafine fiber'**:ab,ti OR **'fiber, ultrafine'**:ab,ti OR **'airborne particulate matter'**:ab,ti OR **'particulate matter, airborne'**:ab,ti OR **'air pollutants, particulate'**:ab,ti OR **'particulate air pollutants'**:ab,ti OR **'ambient particulate matter'**:ab,ti OR **'particulate matter, ambient'**:ab,ti OR **'ultrafine particulate matter'**:ab,ti OR **'particulate matter, ultrafine'**:ab,ti OR **'ultrafine particles'**:ab,ti OR **'particles, ultrafine'**:ab,ti OR **'ultrafine particle'**:ab,ti OR **'particle, ultrafine'**:ab,ti	6,798
**#15**	**#1** OR **#2**	206,332
**#16**	**#3** OR **#4**	98,760
**#17**	**#5** OR **#6**	17,336
**#18**	**#7** OR **#8**	17,298
**#19**	**#9** OR **#10**	44.222
**#20**	**#11** OR **#12**	33,747
**#21**	**#13** OR **#14**	59,304
**#22**	**#15** OR **#16** OR **#17** OR **#18** OR **#19** OR **#20** OR **#21**	291,789
**#23**	**'semen analysis'**/exp	23,279
**#24**	**'semen analyses'**:ab,ti OR **'semen quality analysis'**:ab,ti OR **'analyses, semen quality'**:ab,ti OR **'analysis, semen quality'**:ab,ti OR **'quality analyses, semen'**:ab,ti OR **'semen quality analyses'**:ab,ti OR **'semen quality'**:ab,ti OR **'qualities, semen'**:ab,ti OR **'quality, semen'**:ab,ti OR **'semen qualities'**:ab,ti	8,386
**#25**	**'sperm count'**/exp	16,382
**#26**	**'count, sperm'**:ab,ti OR **'counts, sperm'**:ab,ti OR **'sperm counts'**:ab,ti OR **'sperm number'**:ab,ti OR **'number, sperm'**:ab,ti OR **'numbers, sperm'**:ab,ti OR **'sperm numbers'**:ab,ti	23,306
**#27**	**'spermatozoon motility'**/exp	30,566
**#28**	**'motilities, sperm'**:ab,ti OR **'motility, sperm'**:ab,ti OR **'sperm motilities'**:ab,ti	929
**#29**	**#23** OR **#24**	27,507
**#30**	**#25** OR **#26**	34,274
**#31**	**#27** OR **#28**	30,767
**#32**	**#29** OR **#30** OR **#31**	66,729
**#33**	**#22** AND **#32**	337

The detailed search strategy for database 4: Cochrane Library

#1	MeSH descriptor: [Air Pollution] explode all trees	MeSH		686
#2	(Air Pollutions OR Pollution, Air OR Air Quality):ti,ab,kw (Word variations have been searched)	S	Limits	3744
#3	#1 OR #2		Limits	4011
#4	MeSH descriptor: [Air Pollutants] explode all trees	MeSH		379
#5	(Air Pollutants OR Pollutants, Air OR Air Pollutant OR Pollutant, Air OR Air Pollutants, Environmental OR Environmental Pollutants, Air OR Air Environmental Pollutants OR Pollutants, Air Environmental OR Environmental Air Pollutants OR Pollutants, Environmental Air):ti,ab,kw (Word variations have been searched)	S	Limits	1579
#6	#4 OR #5		Limits	1579
#7	MeSH descriptor: [Sulfur Dioxide] explode all trees	MeSH		48
#8	(Sulfur dioxide OR Sulfurous Anhydride):ti,ab,kw (Word variations have been searched)	S	Limits	126
#9	#7 OR #8		Limits	126
#10	MeSH descriptor: [Nitrogen Dioxide] explode all trees	MeSH		70
#11	(Nitrogen dioxide OR Dioxide, Nitrogen OR Nitrogen Peroxide OR Peroxide, Nitrogen):ti,ab,kw (Word variations have been searched)	S	Limits	453
#12	#10 OR #11		Limits	453
#13	MeSH descriptor: [Carbon Monoxide] explode all trees	MeSH		563
#14	(Carbon monoxide OR Monoxide, Carbon):ti,ab,kw (Word variations have been searched)	S	Limits	2780
#15	#13 OR #14		Limits	2780
#16	MeSH descriptor: [Ozone] explode all trees	MeSH		342
#17	(Ozone OR Tropospheric Ozone OR Ozone, Tropospheric OR Level Ozone, Low OR Low Level Ozone OR Ozone, Low Level OR Ground Level Ozone OR Level Ozone, Ground OR Ozone, Ground Level):ti,ab,kw (Word variations have been searched)	S	Limits	1095
#18	#16 OR #17		Limits	1095
#19	MeSH descriptor: [Particulate Matter] explode all trees	MeSH		914
#20	(Particulate Matter OR Ultrafine Fibers OR Ultrafine Fiber OR Fiber, Ultrafine OR Airborne Particulate Matter OR Particulate Matter, Airborne OR Air Pollutants, Particulate OR Particulate Air Pollutants OR Ambient Particulate Matter OR Particulate Matter, Ambient OR Ultrafine Particulate Matter OR Particulate Matter, Ultrafine OR Ultrafine Particles OR Particles, Ultrafine OR Ultrafine Particle OR Particle, Ultrafine):ti,ab,kw (Word variations have been searched)	S	Limits	778
#21	#19 OR #20		Limits	1452
#22	#3 OR #6 OR #9 OR #12 OR #15 OR #18 OR #21		Limits	8432
#23	MeSH descriptor: [Semen Analysis] explode all trees	MeSH		591
#24	(Semen Analysis OR Semen Analyses OR Semen Quality Analysis OR Analyses, Semen Quality OR Analysis, Semen Quality OR Quality Analyses, Semen OR Semen Quality Analyses OR Semen Quality OR Qualities, Semen OR Quality, Semen OR Semen Qualities):ti,ab,kw (Word variations have been searched)	S	Limits	1573
#25	#23 OR #24		Limits	1855
#26	MeSH descriptor: [Sperm Count] explode all trees	MeSH		356
#27	[illegible] Sperm Numbers):ti,ab,kw (Word variations have been searched)	S	Limits	2842
#28	#26 OR #27		Limits	2842
#29	MeSH descriptor: [Sperm Motility] explode all trees	MeSH		367
#30	(Sperm Motility OR Motilities, Sperm OR Motility, Sperm OR Sperm Motilities):ti,ab,kw (Word variations have been searched)	S	Limits	1455
#31	#29 OR #30		Limits	1455
#32	#25 OR 28 OR #31		Limits	236045
#33	#22 AND #32		Limits	1467

The detailed search strategy for database 5: China National Knowledge Infrastructure databases (CNKI)

（主题：空气污染物（精确））OR（篇关摘：空气污染物 OR 空气污染物, 环境 OR 环境空气污染物 OR 环境空气污染物 OR气态污染物 OR 大气污染物 OR 大气污染 OR 空气污染（精确））OR（主题：颗粒物（精确））OR（篇关摘：颗粒物 OR 大气颗粒物 OR 颗粒空气污染物 OR 大气颗粒物 OR 大气细颗粒物 OR PM2.5 OR PM10（精确））OR（主题：二氧化氮（精确））OR（篇关摘：二氧化氮 OR 过氧化氮 OR NO2（精确））OR（主题：臭氧（精确））OR（篇关摘：臭氧 OR 地面臭氧 OR O3（精确））OR（主题：一氧化碳（精确））OR（篇关摘：一氧化碳 OR CO（精确）） AND （（主题：精液分析（精确））OR（篇关摘：精液分析 OR 精液质量分析 OR 精液质量 OR 精液质量分析（精确））OR（主题：精子计数（精确））OR（篇关摘：精子计数 OR 精子数（精确））OR（主题：精子能动性（精确））OR（篇关摘：精子能动性 OR 精子活力（精确））)

The detailed search strategy for database 6: Wanfang

检索式	检索结果
主题：（空气污染物 OR 空气污染物, 环境 OR 环境空气污染物 OR 环境空气污染物 OR气态污染物 OR 大气污染物 OR 大气污染 OR 空气污染 OR 颗粒物 OR 大气颗粒物 OR 颗粒空气污染物 OR 大气颗粒物 OR 大气细颗粒物 OR PM2.5 OR PM10 OR 二氧化硫 OR 亚硫酐 OR SO2 OR 二氧化氮 OR 过氧化氮 OR NO2 OR臭氧 OR 地面臭氧 OR O3 OR 一氧化碳 OR CO）AND 主题：（精液分析 OR 精液质量分析 OR 精液质量 OR 精液质量分析 OR 精子计数 OR 精子数 OR 精子能动性 OR 精子活力）	316

The detailed search strategy for database 7: VIP

编号	检索结果	检索表达式
1#	84	(((((((((((((((((((((((题名或关键词=空气污染物 OR 题名或关键词=空气污染物, 环境) OR 题名或关键词=环境空气污染物) OR 题名或关键词=环境空气污染物 OR气态污染物) OR 题名或关键词=大气污染物) OR 题名或关键词=大气污染) OR 题名或关键词=空气污染) OR 题名或关键词=颗粒物) OR 题名或关键词=大气颗粒物) OR 题名或关键词=颗粒空气污染物) OR 题名或关键词=大气颗粒物) OR 题名或关键词=大气细颗粒物) OR 题名或关键词=PM2.5) OR 题名或关键词=PM10) OR 题名或关键词=二氧化硫) OR 题名或关键词=亚硫酐) OR 题名或关键词=SO2) OR 题名或关键词=二氧化氮) OR 题名或关键词=过氧化氮) OR 题名或关键词=NO2 OR臭氧) OR 题名或关键词=地面臭氧) OR 题名或关键词=O3) OR 题名或关键词=一氧化碳) OR 题名或关键词=CO) AND (((((((题名或关键词=精液分析 OR 题名或关键词=精液质量分析) OR 题名或关键词=精液质量) OR 题名或关键词=精液质量分析) OR 题名或关键词=精子计数) OR 题名或关键词=精子数) OR 题名或关键词=精子能动性) OR 题名或关键词=精子活力))

Table S1. Search words.

Air pollution	Air Pollutions	Semen Analysis	Semen Analyses
	Pollution, Air		Semen Quality Analysis
	Air Quality		Analyses, Semen Quality
Air Pollutants	Pollutants, Air		Analysis, Semen Quality
	Air Pollutant		Quality Analyses, Semen
	Pollutant, Air		Semen Quality Analyses
	Air Pollutants, Environmental		Semen Quality
	Environmental Pollutants, Air		Qualities, Semen
	Air Environmental Pollutants		Quality, Semen
	Pollutants, Air Environmental		Semen Qualities
	Environmental Air Pollutants	Sperm Count	Count, Sperm
	Pollutants, Environmental Air		Counts, Sperm
Sulfur dioxide	Sulfurous Anhydride		Sperm Counts
Nitrogen dioxide	Dioxide, Nitrogen		Sperm Number
	Nitrogen Peroxide		Number, Sperm
	Peroxide, Nitrogen		Numbers, Sperm
Carbon monoxide	Monoxide, Carbon		Sperm Numbers
Ozone	Tropospheric Ozone	Sperm Motility	Motilities, Sperm
	Ozone, Tropospheric		Motility, Sperm
	Level Ozone, Low		Sperm Motilities
	Low Level Ozone		
	Ozone, Low Level		
	Ground Level Ozone		
	Level Ozone, Ground		
	Ozone, Ground Level		
Particulate Matter	Ultrafine Fibers		
	Ultrafine Fiber		
	Fiber, Ultrafine		
	Airborne Particulate Matter		
	Particulate Matter, Airborne		
	Air Pollutants, Particulate		
	Particulate Air Pollutants		
	Ambient Particulate Matter		
	Particulate Matter, Ambient		
	Ultrafine Particulate Matter		
	Particulate Matter, Ultrafine		
	Ultrafine Particles		
	Particles, Ultrafine		
	Ultrafine Particle		
	Particle, Ultrafine		

Table S2. Excluded studies and corresponding reasons for exclusion.

Study	Study description	Reason for exclusion
Xiong *et al*. (2021)	A prospective cohort study on the effects of air pollutants exposure during 90 days before sampling on the reproductive endocrine system of male college students.	No relevant effect size. .
Cheng *et al*. (2022)	A cross-sectional study on the association of long-term exposure to air pollution and semen quality and sperm DNA methylation.	No relevant exposure period.
Chen *et al*. (2020)	A retrospective study on the effects of air pollutants exposure on semen quality.	Unknown exposure period.
Sun *et al*. (2020)	A study on the exposure-lag-response relationship between semen quality and weekly air pollution exposure.	No relevant exposure period.
Wu *et al*. (2021)	A retrospective cohort study on the association between $PM_{2.5}$ constituents and the semen quality.	Missing effect size data.
Zhang *et al*. (2020)	A retrospective cohort study on the effects of air pollution on sperm quality in terms of the	No relevant outcomes.

	perspectives of oxidative stress and relative molecular damage.	
Song et al. (2019)	A review on the relationship between ambient air pollution and human sperm quality.	Review article.
Yang *et al*. (2021)	A retrospective observational study on the effects of air pollution exposure on semen quality.	Missing effect size data.
Zhang *et al*. (2020)	A systematic review and meta-analysis on air pollution exposure on semen quality.	Review article.
Qian *et al*. (2022)	A systematic review and meta-analysis on air pollution exposure on semen quality in adults.	Review article.
Guo *et al*. (2021)	A study on the association between chemical components of fine particulate matter with emergency department visits in Guangzhou, China.	No relevant effect size.
Zhang *et al*. (2019)	A protocol for updated systematic review and meta-analysis on association between outdoor air pollution and semen quality.	Protocol.
Deng *et al*. (2016)	A systematic review and meta-analysis on the association between air pollution and sperm quality.	Review article.
Wu *et al*. (2018)	A study on the association between long-term exposure to ambient fine particulate matter and semen quality.	No relevant exposure period.

References

Xiong Y, Wang FR, Chen Q, Yang H, Zhou WZ, Zhang D, Cao J, Zhou NY. Association of exposure to environmental gaseous in Chongqing with semen quality and sex hormone levels in Chongqing young men. *Acta Acadiemiae Midicinae Militaris Tertiae* 2021;4**3(1)**:10-16.

Cheng Y, Tang Q, Lu Y, Li M, Zhou Y, Wu P, Li J, Pan F, Han X, Chen M, Lu C, Wang X, Wu W, Xia Y. Semen quality and sperm DNA methylation in relation to long-term exposure to air pollution in fertile men: A cross-sectional study. Environ Pollut. 2022 May 1;300:118994.

Chen YA, Chang YK, Su YR, Chang HC. Ambient sulfur dioxide could have an impact on testicular volume from a observational study on a population of infertile male. BMC Urol. 2020 Oct 2;20(1):149.

Sun S, Zhao J, Cao W, Lu W, Zheng T, Zeng Q. Identifying critical exposure windows for ambient air pollution and semen quality in Chinese men. Environ Res. 2020 Oct;189:109894.

Wu H, Yu X, Wang Q, Zeng Q, Chen Y, Lv J, Wu Y, Zhou H, Zhang H, Liu M, Zheng M, Zhao Q, Guo P, Feng W, Zhang X, Tian L. Beyond the mean: Quantile regression to differentiate the distributional effects of ambient $PM_{2.5}$ constituents on sperm quality among men. Chemosphere. 2021 Dec;285:131496.

Zhang G, Jiang F, Chen Q, Yang H, Zhou N, Sun L, Zou P, Yang W, Cao J, Zhou Z, Ao L. Associations of ambient air pollutant exposure with

seminal plasma MDA, sperm mtDNA copy number, and mtDNA integrity. Environ Int. 2020 Mar;136:105483.

Song MN, Li JF, Diao CY, Chang B. Analysis on relationship between ambient air pollution and human sperm quality. Journal of environmental hygiene, 2019, 9(3): 294-298.

Yang T, Deng L, Sun B, Zhang S, Xian Y, Xiao X, Zhan Y, Xu K, Buonocore JJ, Tang Y, Li F, Qiu Y. Semen quality and windows of susceptibility: A case study during COVID-19 outbreak in China. Environ Res. 2021 Jun;197:111085.

Zhang J, Cai Z, Ma C, Xiong J, Li H. Impacts of Outdoor Air Pollution on Human Semen Quality: A Meta-Analysis and Systematic Review. Biomed Res Int. 2020 Apr 28;2020:7528901.

Qian H, Xu Q, Yan W, Fan Y, Li Z, Tao C, Zhang F, Lu C. Association between exposure to ambient air pollution and semen quality in adults: a meta-analysis. Environ Sci Pollut Res Int. 2022 Feb;29(7):10792-10801.

Guo P, Wu H, Chen YL, Lv JY, Shi TX, Liu PD, Wu Y, Zhou HW, Zhang HF, Liu M, Zheng MR, Feng WR. Associations of chemical components of fine particulate matter with emergency department visits in Guangzhou, China. Atmospheric Environment, 2020, 246:118097.

Zhang J, Cai Z, Yang B, Li H. Association between outdoor air pollution and semen quality: Protocol for an updated systematic review and meta-analysis. Medicine (Baltimore). 2019 May;98(20):e15730.

Deng Z, Chen F, Zhang M, Lan L, Qiao Z, Cui Y, An J, Wang N, Fan Z, Zhao X, Li X. Association between air pollution and sperm quality: A systematic review and meta-analysis. Environ Pollut. 2016 Jan;208(Pt B):663-9.

Wu L, Zhang H, Peng Z, Fan CG, Jin L, Shi YM, Zhou Y, Liu YW. Association between long-term exposure to ambient fine particulate matter and semen quality. J of Pub Health and Prev Med, 2018, 29(6): 13-16.

Table S3. GRADE assessment of cumulative evidence for the relationship between air pollution exposure and semen quality.

Pollution type	Semen parameters	Exposure periods	Pooled effect estimates	Quality assessment					Quality of evidence
				Risk of bias[a]	Inconsistency[b]	Indirectness[c]	Imprecision[d]	Publication bias [e]	
$PM_{2.5}$	Sperm concentration	Lag 0-9 days	-0.002 (-0.011, 0.007)	Not serious	Not serious	Direct	Serious	Undetected	Low
		Lag10-14 days	-0.004 (-0.009, 0.002)	Not serious	Not serious	Direct	Serious	Undetected	Low
		Lag 70-90 days	0.004 (-0.038, 0.047)	Not serious	Very serious	Direct	Serious	Undetected	Very low
		Lag 0-90 days	0.282 (-0.081, 0.645)	Not serious	Very serious	Direct	Serious	Undetected	Very low
	Total sperm count	Lag 0-9 days	-0.007 (-0.017, 0.002)	Not serious	Not Serious	Direct	Serious	Undetected	Low
		Lag10-14 days	-0.009 (-0.015, -0.003)	Not serious	Not serious	Direct	Not serious	Undetected	Moderate
		Lag 70-90 days	-0.014 (-0.035, 0.006)	Not serious	Serious	Direct	Serious	Undetected	Very low
		Lag 0-90 days	-0.030 (-0.065, 0.004)	Not serious	Very serious	Direct	Serious	Undetected	Very low
	Total motility	Lag 0-9 days	-0.014 (-0.043, 0.014)	Not serious	Very serious	Direct	Serious	Undetected	Very low
		Lag10-14 days	-0.028 (-0.056, -0.001)	Not serious	Very serious	Direct	Not serious	Undetected	Very low
		Lag 70-90 days	-0.073 (-0.125, -0.021)	Not serious	Very serious	Direct	Not serious	Undetected	Very low
		Lag 0-90 days	-0.164 (-0.252, -0.076)	Not serious	Very serious	Direct	Not serious	Undetected	Very low
	Progressive motility	Lag 0-9 days	0.007 (-0.014, 0.027)	Not serious	Very serious	Direct	Serious	Undetected	Very low
		Lag10-14 days	-0.013 (-0.026, -0.000)	Not serious	Serious	Direct	Not serious	Undetected	Low
		Lag 70-90 days	-0.029 (-0.074, 0.016)	Not serious	Very serious	Direct	Serious	Undetected	Very low
		Lag 0-90 days	-0.051 (-0.109, 0.006)	Not serious	Very serious	Direct	Serious	Undetected	Very low
PM_{10}	Sperm concentration	Lag 0-9 days	-0.000 (-0.006, 0.005)	Not serious	Not serious	Direct	Serious	Undetected	Low
		Lag10-14 days	-0.002 (-0.005, 0.001)	Not serious	Not serious	Direct	Serious	Undetected	Low
		Lag 70-90 days	-0.006 (-0.033, 0.020)	Not serious	Very serious	Direct	Serious	Undetected	Very low
		Lag 0-90 days	-0.036 (-0.065, -0.008)	Not serious	Very serious	Direct	Not serious	Undetected	Very low
	Total sperm count	Lag 0-9 days	-0.002 (-0.010, 0.005)	Not serious	Serious	Direct	Serious	Undetected	Very low
		Lag10-14 days	-0.004 (-0.009, 0.000)	Not serious	Not serious	Direct	Serious	Undetected	Low

Table S3. ***(continued)***

Pollution type	Semen parameters	Exposure periods	Pooled effect estimates	Quality assessment					Quality of evidence
				Risk of bias[a]	Inconsistency[b]	Indirectness[c]	Imprecision[d]	Publication bias [e]	
		Lag 70-90 days	-0.013 (-0.021, -0.005)	Not serious	Not serious	Direct	Not serious	Undetected	Moderate
		Lag 0-90 days	-0.037 (-0.060, -0.014)	Not serious	Very serious	Direct	Not serious	Undetected	Very low
	Total motility	Lag 0-9 days	-0.005 (-0.019, 0.008)	Not serious	Very serious	Direct	Serious	Undetected	Very low
		Lag10-14 days	-0.010 (-0.022, 0.002)	Not serious	Very serious	Direct	Serious	Undetected	Very low
		Lag 70-90 days	-0.034 (-0.070, 0.001)	Not serious	Very serious	Direct	Serious	Undetected	Very low
		Lag 0-90 days	-0.067 (-0.110, -0.025)	Not serious	Very serious	Direct	Not serious	Undetected	Very low
	Progressive motility	Lag 0-9 days	0.005 (-0.005, 0.015)	Not serious	Serious	Direct	Serious	Undetected	Very low
		Lag10-14 days	-0.006 (-0.014, 0.002)	Not serious	Serious	Direct	Serious	Undetected	Very low
		Lag 70-90 days	0.002 (-0.024, 0.029)	Not serious	Very serious	Direct	Serious	Undetected	Very low
		Lag 0-90 days	-0.030 (-0.068, 0.007)	Not serious	Very serious	Direct	Serious	Undetected	Very low
SO_2	Sperm concentration	Lag 0-9 days	-0.071 (-0.494, 0.353)	Not serious	Very serious	Direct	Serious	Undetected	Very low
		Lag10-14 days	-0.205 (-0.477, 0.067)	Not serious	Very serious	Direct	Serious	Undetected	Very low
		Lag 70-90 days	0.418 (-0.292, 1.129)	Not serious	Very serious	Direct	Serious	Undetected	Very low
		Lag 0-90 days	-0.117 (-0.387, 0.153)	Not serious	Very serious	Direct	Not serious	Undetected	Very low
	Total sperm count	Lag 0-9 days	-0.105 (-0.348, 0.137)	Not serious	Serious	Direct	Serious	Undetected	Very low
		Lag10-14 days	-0.261 (-0.537, 0.015)	Not serious	Serious	Direct	Serious	Undetected	Very low
		Lag 70-90 days	-0.125 (-0.260, 0.010)	Not serious	Serious	Direct	Serious	Undetected	Very low
		Lag 0-90 days	-0.099 (-0.169, -0.030)	Not serious	Serious	Direct	Not serious	Undetected	Low
	Total motility	Lag 0-9 days	-0.131 (-0.153, -0.109)	Not serious	Not serious	Direct	Not serious	Undetected	Moderate
		Lag10-14 days	-0.122 (-0.201, -0.043)	Not serious	Not serious	Direct	Not serious	Undetected	Moderate
		Lag 70-90 days	-0.130 (-0.339, 0.079)	Not serious	Serious	Direct	Serious	Undetected	Very low
		Lag 0-90 days	-0.192 (-0.362, -0.022)	Not serious	Serious	Direct	Not serious	Undetected	Low

Table S3. *(continued)*

Pollution type	Semen parameters	Exposure periods	Pooled effect estimates	Quality assessment					Quality of evidence
				Risk of bias[a]	Inconsistency[b]	Indirectness[c]	Imprecision[d]	Publication bias [e]	
	Progressive motility	Lag 0-9 days	0.130 (-0.088, 0.348)	Not serious	Very serious	Direct	Serious	Undetected	Very low
		Lag10-14 days	-0.012 (-0.121, 0.097)	Not serious	Serious	Direct	Serious	Undetected	Very low
		Lag 70-90 days	0.006 (-0.204, 0.217)	Not serious	Very serious	Direct	Serious	Undetected	Very low
		Lag 0-90 days	-0.046 (-0.215, 0.123)	Not serious	Very serious	Direct	Not serious	Undetected	Very low
NO_2	Sperm concentration	Lag 0-9 days	0.001 (-0.035, 0.038)	Not serious	Serious	Direct	Serious	Undetected	Very low
		Lag10-14 days	-0.000 (-0.029, 0.029)	Not serious	Serious	Direct	Serious	Undetected	Very low
		Lag 70-90 days	0.074 (-0.076, 0.223)	Not serious	Very serious	Direct	Serious	Undetected	Very low
		Lag 0-90 days	0.042 (-0.118, 0.202)	Not serious	Very serious	Direct	Serious	Undetected	Very low
	Total sperm count	Lag 0-9 days	-0.006 (-0.031, 0.019)	Not serious	Not serious	Direct	Serious	Undetected	Low
		Lag10-14 days	-0.009 (-0.035, 0.016)	Not serious	Not serious	Direct	Serious	Undetected	Low
		Lag 70-90 days	-0.015 (-0.028, -0.002)	Not serious	Not serious	Direct	Not serious	Undetected	Moderate
		Lag 0-90 days	-0.020 (-0.035, -0.004)	Not serious	Not serious	Direct	Not serious	Undetected	Moderate
	Total motility	Lag 0-9 days	0.007 (-0.003, 0.017)	Not serious	Not serious	Direct	Serious	Undetected	Low
		Lag10-14 days	0.004 (-0.007, 0.016)	Not serious	Not serious	Direct	Serious	Undetected	Low
		Lag 70-90 days	-0.013 (-0.029, 0.004)	Not serious	Not serious	Direct	Serious	Undetected	Low
		Lag 0-90 days	-0.013 (-0.028, 0.002)	Not serious	Not serious	Direct	Serious	Undetected	Low
	Progressive motility	Lag 0-9 days	0.043 (0.007, 0.079)	Not serious	Very serious	Direct	Not serious	Undetected	Very low
		Lag10-14 days	0.008 (-0.014, 0.029)	Not serious	Serious	Direct	Serious	Undetected	Very low
		Lag 70-90 days	-0.002 (-0.043, 0.039)	Not serious	Serious	Direct	Serious	Undetected	Very low
		Lag 0-90 days	0.057 (-0.024, 0.139)	Not serious	Very serious	Direct	Serious	Undetected	Very low
CO	Sperm concentration	Lag 0-9 days	0.002 (-0.006, 0.011)	Not serious	Serious	Direct	Serious	Undetected	Very low
		Lag10-14 days	-0.001 (-0.003, 0.001)	Not serious	Not serious	Direct	Serious	Undetected	Low

Table S3. ***(continued)***

Pollution type	Semen parameters	Exposure periods	Pooled effect estimates	Quality assessment					Quality of evidence
				Risk of bias[a]	Inconsistency[b]	Indirectness[c]	Imprecision[d]	Publication bias [e]	
		Lag 70-90 days	0.026 (-0.004, 0.056)	Not serious	Very serious	Direct	Serious	Undetected	Very low
		Lag 0-90 days	0.016 (-0.006, 0.038)	Not serious	Very serious	Direct	Serious	Undetected	Very low
	Total sperm count	Lag 0-9 days	-0.004 (-0.014, 0.005)	Not serious	Serious	Direct	Serious	Undetected	Very low
		Lag10-14 days	-0.002 (-0.010, 0.005)	Not serious	Serious	Direct	Serious	Undetected	Very low
		Lag 70-90 days	-0.008 (-0.023, 0.007)	Not serious	Very serious	Direct	Serious	Undetected	Very low
		Lag 0-90 days	-0.006 (-0.020, 0.008)	Not serious	Very serious	Direct	Serious	Undetected	Very low
	Total motility	Lag 0-9 days	-0.007 (-0.017, 0.003)	Not serious	Very serious	Direct	Serious	Undetected	Very low
		Lag10-14 days	-0.007 (-0.015, 0.000)	Not serious	Very serious	Direct	Serious	Undetected	Very low
		Lag 70-90 days	-0.012 (-0.026, 0.002)	Not serious	Very serious	Direct	Serious	Undetected	Very low
		Lag 0-90 days	-0.010 (-0.025, 0.004)	Not serious	Very serious	Direct	Serious	Undetected	Very low
	Progressive motility	Lag 0-9 days	0.003 (-0.003, 0008)	Not serious	Very serious	Direct	Serious	Undetected	Very low
		Lag10-14 days	-0.001 (-0.002, 0.001)	Not serious	Not serious	Direct	Serious	Undetected	Low
		Lag 70-90 days	0.001 (-0.005, 0.007)	Not serious	Serious	Direct	Serious	Undetected	Very low
		Lag 0-90 days	0.005 (-0.005, 0.015)	Not serious	Very serious	Direct	Serious	Undetected	Very low
O_3	Sperm concentration	Lag 0-9 days	-0.012 (-0.049, 0.024)	Not serious	Very serious	Direct	Serious	Undetected	Very low
		Lag10-14 days	-0.021 (-0.047, 0.004)	Not serious	Serious	Direct	Serious	Undetected	Very low
		Lag 70-90 days	0.001 (-0.008, 0.009)	Not serious	Not serious	Direct	Serious	Undetected	Low
		Lag 0-90 days	-0.027 (-0.113, 0.058)	Not serious	Serious	Direct	Serious	Undetected	Very low
	Total sperm count	Lag 0-9 days	-0.005 (-0.030, 0.020)	Not serious	Serious	Direct	Serious	Undetected	Very low
		Lag10-14 days	-0.013 (-0.034, 0.008)	Not serious	Serious	Direct	Serious	Undetected	Very low
		Lag 70-90 days	0.006 (-0.003, 0.015)	Not serious	Not serious	Direct	Serious	Undetected	Low
		Lag 0-90 days	-0.021 (-0.080, 0.039)	Not serious	Not serious	Direct	Serious	Undetected	Low

Table S3. ***(continued)***

Pollution type	Semen parameters	Exposure periods	Pooled effect estimates	Quality assessment					Quality of evidence
				Risk of bias[a]	Inconsistency[b]	Indirectness[c]	Imprecision[d]	Publication bias [e]	
	Total motility	Lag 0-9 days	0.003 (-0.010, 0.017)	Not serious	Serious	Direct	Serious	Undetected	Very low
		Lag10-14 days	0.003 (-0.007, 0.013)	Not serious	Serious	Direct	Serious	Undetected	Very low
		Lag 70-90 days	0.008 (-0.004, 0.021)	Not serious	Not serious	Direct	Serious	Undetected	Low
		Lag 0-90 days	0.017 (-0.004, 0.038)	Not serious	Not serious	Direct	Serious	Undetected	Low
	Progressive motility	Lag 0-9 days	-0.003 (-0.015, 0.009)	Not serious	Serious	Direct	Serious	Undetected	Very low
		Lag10-14 days	-0.003 (-0.009, 0.003)	Not serious	Not serious	Direct	Serious	Undetected	Low
		Lag 70-90 days	-0.003 (-0.010, 0.004)	Not serious	Not serious	Direct	Serious	Undetected	Low
		Lag 0-90 days	-0.006 (-0.016, 0.003)	Not serious	Not serious	Direct	Serious	Undetected	Low

a. Risk of bias was evaluated based on the inclusion criteria.

b. Inconsistency was evaluated based on I^2 statistics: I^2<50%, not serious; 50%≤ I^2 <80%, serious; I^2 ≥80%, very serious.

c. Indirectness: we considered no indirectness if the evidence directly came from the studies related to the topic of meta-analysis,.

d. Imprecision was assessed based on the 95% CI of β.

e. Publication bias was assessed based on Egger's regression test for asymmetry.

Table S4. Pooled associations between semen quality and pollutants exposure (per 10 μg/m^3 increment) during 0-90 lag days.

$PM_{2.5}$	Sperm concentration	Overall	15	0.282 (-0.081, 0.645)	0.128	17304.29	99.9	<0.001
		Cross-sectional	2	1.027 (-0.999, 3.052)	0.320	3045.57	99.9	<0.001
		Cohort	13	-0.032 (-0.088, 0.024)	0.264	175.14	93.1	<0.001
		Northern China	2	2.362 (-2.329, 7.053)	0. 324	97.06	99.0	<0.001
		Southern China	13	0.052 (-0.341, 0.444)	0.796	17194.92	99. 9	<0.001
		Estimating model	9	0.201 (-0.403, 0.805)	0.515	16857.04	99. 9	<0.001
		Monitoring station	6	0.242 (-0.090, 0.575)	0.153	132.67	96. 2	<0.001
	Total sperm count	Overall	12	-0.030 (-0.065, 0.004)	0.086	77.25	85.8	<0.001
		Cross-sectional	1	-0.021 (-0.089, 0.047)	0.542	NA	NA	NA
		Cohort	11	-0.033 (-0.070, 0.005)	0.089	76.76	88.3	<0.001
		Northern China	1	-0.021 (-0.089, 0.047)	0.542	NA	NA	NA
		Southern China	11	-0.031 (-0.068, 0.006)	0.098	77.08	87.0	<0.001
		Estimating model	8	-0.049 (-0.073, -0.025)	<0.001	16.06	56.4	0. 025
		Monitoring station	4	0.051 (-0.046, 0.148)	0.302	5.73	47.6	0. 126
	Total motility	Overall	12	-0.164 (-0.252, -0.076)	<0.001	508.06	97.8	<0.001
		Cross-sectional	2	0.120 (-0.255, 0.495)	0. 531	3.31	69.8	0. 069
		Cohort	10	-0.206 (-0.305, -0.107)	<0.001	498.52	98.2	<0.001
		Northern China	1	-0.021 (-0.064, 0.022)	0. 333	NA	NA	NA
		Southern China	11	-0.184 (-0.282, -0.087)	<0.001	502.77	98.0	<0.001
		Estimating model	9	-0.032 (-0.086, 0.021)	0. 240	96.70	91.7	<0.001
		Monitoring station	3	-0.798 (-1.887, 0.291)	0. 151	410.20	99.5	<0.001
	Progressive motility	Overall	13	-0.051 (-0.109, 0.006)	0.081	174.42	93.1	<0.001
		Cross-sectional	2	-0.036 (-0.083, 0.012)	0.144	0.07	0.0	0. 792

Table S4. (*continued*)

Pollution type	Semen parameters	Groups	No. of studies	Summary β (95% CI)	*P*	Test of heterogeneity X^2	I^2(%)	*P*
		Cohort	11	-0.053 (-0.118, 0.011)	0. 106	173.87	94.2	<0.001
		Northern China	2	0.281 (-0.351, 0.913)	0. 383	46.05	97.8	<0.001
		Southern China	11	-0.096 (-0.154, -0.039)	<0.001	121.67	91.8	<0.001
		Estimating model	8	-0.045 (-0.089, -0.001)	0. 043	72.50	90.3	<0.001
		Monitoring station	5	-0.121 (-0.731, 0.489)	0. 697	101.80	96.1	<0.001
PM_{10}	Sperm concentration	Overall	13	-0.044 (-0.094, 0.006)	0.087	115.65	89.6	<0.001
		Cross-sectional	2	-0.115 (-0.509, 0.280)	0.569	1.95	48.7	0. 163
		Cohort	11	-0.047 (-0.106, 0.012)	0.118	113.65	91.2	<0.001
		Northern China	2	1.462 (-1.457, 4.382)	0. 326	53.85	98.1	<0.001
		Southern China	11	-0.061 (-0.105, -0.016)	0.008	61.34	83.7	<0.001
		Estimating model	6	-0.042 (-0.075, -0.010)	0.010	12.28	59.3	0. 031
		Monitoring station	7	0.130 (-0.154, 0.414)	0.369	91.94	93.5	<0.001
	Total sperm count	Overall	11	-0.037 (-0.060, -0.014)	0.002	71.34	86.0	<0.001
		Cross-sectional	2	-0.401 (-1.604, 0.802)	0.514	2.31	56.8	0. 128
		Cohort	9	-0.040 (-0.065, -0.015)	0.002	69.0	88.4	<0.001
		Northern China	1	-0.014 (-0.054, 0.026)	0. 487	NA	NA	NA
		Southern China	10	-0.041 (-0.066, -0.016)	0.002	71.30	87.4	<0.001
		Estimating model	7	-0.039 (-0.056, -0.022)	<0.001	12.00	50.0	0. 062
		Monitoring station	4	-0.057 (-0.189, 0.074)	0.392	8.29	63.8	0. 040
	Total motility	Overall	11	-0.067 (-0.110, -0.025)	0. 002	148.41	93.3	<0.001
		Cross-sectional	2	-0.147 (-0.470, 0.175)	0.370	5.15	80.6	0.023
		Cohort	9	-0.073 (-0.124, -0.023)	0.005	137.47	94.2	<0.001
		Northern China	1	-0.012 (-0.036, 0.012)	0.317	NA	NA	NA

Table S4. (*continued*)

Pollution type	Semen parameters	Groups	No. of studies	Summary β (95% CI)	*P*	Test of heterogeneity		
						X^2	I^2(%)	*P*
		Southern China	10	-0.081 (-0.131, -0.030)	0. 002	141.70	93.6	<0.001
		Estimating model	7	-0.021 (-0.049, 0.007)	0.137	50.75	88.2	<0.001
		Monitoring station	4	-0.317 (-0.657, 0.024)	0.068	11.69	74.3	0. 009
	Progressive motility	Overall	14	-0.030 (-0.068, 0.007)	0. 116	141.89	90.8	<0.001
		Cross-sectional	3	0.083 (-0.331, 0.497)	0. 695	61.96	96.8	<0.001
		Cohort	11	-0.051 (-0.085, -0.016)	0. 004	75.97	86.8	<0.001
		Northern China	2	0.269 (-0.310, 0.848)	0. 362	54.14	98.2	<0.001
		Southern China	12	-0.057 (-0.093, -0.022)	0. 002	82.29	86.6	<0.001
		Estimating model	7	-0.028 (-0.057, 0.000)	0. 052	53.61	88.8	<0.001
		Monitoring station	7	0.010 (-0.273, 0.294)	0. 944	86.81	93.1	<0.001
SO_2	Sperm concentration	Overall	9	-0.117 (-0.387, 0.153)	0.396	163.00	95.1	<0.001
		Cross-sectional	2	-1.534 (-2.295, -0.773)	<0.001	0.19	0.0	0. 660
		Cohort	7	0.020 (-0.253, 0.294)	0.883	148.15	96.0	<0.001
		Northern China	2	18.596 (-20.034, 57.226)	0. 345	126.79	99.2	<0.001
		Southern China	7	-0.072 (-0.187, 0.043)	0.222	23.22	74.2	0.001
		Estimating model	5	-0.054 (-0084, -0.023)	0. 001	4.34	7.7	0. 363
		Monitoring station	4	6.073 (1.881, 10.264)	0.005	157.53	98.1	<0.001
	Total sperm count	Overall	6	-0.099 (-0.169, -0.030)	0.005	9.99	50.0	0. 075
	Total motility	Overall	6	-0.192 (-0.362, -0.022)	0.027	17.59	71.6	0. 004
	Progressive motility	Overall	9	-0.046 (-0.215, 0.123)	0.590	84.94	90.6	<0.001
		Cross-sectional	2	-0.250 (-0.509, 0.008)	0.058	0.15	0.0	0. 700

Table S4. (*continued*)

Pollution type	Semen parameters	Subgroups	No. of studies	Summary β (95% CI)	*P*	Test of heterogeneity		
						X^2	I^2(%)	*P*
		Cohort	7	-0.002 (-0.189, 0.184)	0.980	81.17	92.6	<0.001
		Northern China	2	2.261 (-3.265, 7.788)	0. 423	26.04	96.2	<0.001
		Southern China	7	-0.066 (-0.150, 0.018)	0.124	17.29	65.3	0. 008
		Estimating model	5	-0.038 (-0.119, 0.043)	0.359	12.60	68.3	0. 013
		Monitoring station	4	1.006 (-0.620, 2.631)	0.225	71.97	95.8	<0.001
NO_2	Sperm concentration	Overall	9	0.042 (-0.118, 0.202)	0.610	102.03	92.2	<0.001
	Total sperm count	Overall	6	-0.020 (-0.035, -0.004)	0.014	2.71	0.00	0. 744
	Total motility	Overall	6	-0.013 (-0.028, 0.002)	0.097	4.41	0.0	0.492
	Progressive motility	Overall	9	0.057 (-0.024, 0.139)	0.169	60.98	86.9	0.005
		Cross-sectional	3	0.566 (-0.095, 1.228)	0.093	48.40	95.9	<0.001
		Cohort	6	-0.008 (-0.050, 0.034)	0.699	11.13	55.1	0. 049
		Northern China	2	0.751 (-0.897, 2.399)	0.372	42.34	97.6	<0.001
		Southern China	7	0.009 (-0.044, 0.061)	0.747	18.64	67.8	0. 005
		Estimating model	5	-0.014 (-0.044, 0.015)	0.349	6.41	37.6	0. 170
		Monitoring station	4	0.644 (0.007, 1.282)	0.048	24.42	87.7	<0.001
CO	Sperm concentration	Overall	7	0.016 (-0.006, 0.038)	0.150	112.71	94.7	<0.001
		Cross-sectional	1	-0.122 (-0.295, 0.051)	0.167	NA	NA	NA
		Cohort	6	0.018 (-0.004, 0.040)	0.106	110.78	95.5	<0.001
		Northern China	2	0.084 (-0.302, 0.471)	0.669	18.22	94.5	<0.001
		Southern China	5	-0.004 (-0.012, 0.005)	0.385	14.46	72.3	0. 006
		Estimating model	5	-0.003 (-0.010, 0.004)	0.420	10.31	61.2	0. 035
		Monitoring station	2	0.070 (-0.334, 0.474)	0.734	42.95	97.7	<0.001
	Total sperm count	Overall	5	-0.006 (-0.020, 0.008)	0.410	23.91	83.3	<0.001

Table S4. (*continued*)

Pollution type	Semen parameters	Subgroups	No. of studies	Summary β (95% CI)	*P*	Test of heterogeneity X^2	I^2(%)	*P*
	Total motility	Overall	5	-0.010 (-0.025, 0.004)	0.174	76.97	94.8	<0.001
	Progressive motility	Overall	7	0.005 (-0.005, 0.015)	0. 299	55.61	89.2	<0.001
		Cross-sectional	1	-0.140 (-0.262, -0.019)	0. 024	NA	NA	NA
		Cohort	6	0.006 (-0.003, 0.015)	0. 205	50.34	90.1	<0.001
		Northern China	2	-0.041 (-0.212, 0.130)	0. 637	8.01	87.5	0. 005
		Southern China	5	0.000 (-0.002, 0.003)	0. 772	4.07	1.7	0. 397
		Estimating model	5	0.001 (-0.004, 0.006)	0. 743	8.73	54.2	0. 068
		Monitoring station	2	0.015 (-0.028, 0.058)	0. 483	11.0	90.9	0. 001
O_3	Sperm concentration	Overall	8	-0.027 (-0.113, 0.058)	0.527	27.01	74.1	<0.001
		Cross-sectional	1	-0.080 (-0.278, 0.118)	0.428	NA	NA	NA
		Cohort	7	-0.022 (-0.116, 0.072)	0.650	26.57	77.4	<0.001
		Northern China	2	-1.138 (-3.348, 1.072)	0.313	14.78	93.2	<0.001
		Southern China	6	-0.011 (-0.068, 0.046)	0.705	10.41	52.0	0. 064
		Estimating model	5	-0.015 (-0.048, 0.017)	0.354	5.10	21.5	0. 278
		Monitoring station	3	-1.432 (-4.332, 1.469)	0.333	16.63	88.0	<0.001
	Total sperm count	Overall	6	-0.021 (-0.080, 0.039)	0. 496	8.53	41.4	0. 129
	Total motility	Overall	4	0.017 (-0.004, 0.038)	0. 104	3.05	1.6	0. 384
	Progressive motility	Overall	7	-0.006 (-0.016, 0.003)	0.193	7.86	23.6	0.249

Abbreviations: $PM_{2.5}$, particulate matter with the diameter ≤2.5 μm; PM_{10}, particulate matter with diameter ≤10 μm; SO_2, sulfur dioxide; NO_2, nitrogen dioxide; CO, carbon monoxide; O_3, ozone; β, correlation coefficient.

Table S5. Pooled association between semen quality and pollutants exposure (per 10 μg/m^3 increment) during 0-9, 10-14, and 70-90 lag days.

Pollution type	Semen parameters	Subgroups	No. of studies	Summary β (95% CI)	*P*	Test of heterogeneity		
						X^2	I^2(%)	*P*
$PM_{2.5}$	Sperm concentration							
		Lag 0-9 days	8	-0.002 (-0.011, 0.007)	0.675	10.67	34.4	0. 154
`		Lag 10-14 days	8	-0.004 (-0.009, 0.002)	0.186	5.02	0.0	0. 657
		Lag 70-90 days	8	0.004 (-0.038, 0.047)	0.837	116.34	94.0	<0.001
	Total sperm count							
		Lag 0-9 days	7	-0.007 (-0.017, 0.002)	0.128	10.24	41.4	0. 115
		Lag 10-14 days	7	-0.009 (-0.015, -0.003)	0.005	8.00	25.0	0. 238
		Lag 70-90 days	7	-0.014 (-0.035, 0.006)	0.175	23.39	74.3	0.001
	Total motility							
		Lag 0-9 days	8	-0.014 (-0.043, 0.014)	0. 331	59.58	88.3	<0.001
		Lag 10-14 days	8	-0.028 (-0.056, -0.001)	0. 046	74.85	90.6	<0.001
		Lag 70-90 days	8	-0.073 (-0.125, -0.021)	0. 006	143.98	95.1	<0.001
	Progressive motility							
		Lag 0-9 days	9	0.007 (-0.014, 0.027)	0.535	40.90	80.4	<0.001
		Lag 10-14 days	9	-0.013 (-0.026, -0.000)	0050	22.84	65.0	0. 004
		Lag 70-90 days	9	-0.029 (-0.074, 0.016)	0.205	116.33	93.1	<0.001
PM_{10}	Sperm concentration							
		Lag 0-9 days	8	-0.000 (-0.006, 0.005)	0.887	10.99	36.3	0. 139
		Lag 10-14 days	8	-0.002 (-0.005, 0.001)	0.125	2.27	0.0	0. 944
		Lag 70-90 days	8	-0.006 (-0.033, 0.020)	0.628	97.25	92.8	<0.001
	Total sperm count							
		Lag 0-9 days	7	-0.002 (-0.010, 0.005)	0.532	12.93	53.6	0. 044
		Lag 10-14 days	7	-0.004 (-0.009, 0.000)	0.052	8.19	26.8	0. 224

Table S5. (*continued*)

Pollution type	Semen parameters	Subgroups	No. of studies	Summary β (95% CI)	*P*	Test of heterogeneity		
						X^2	I^2(%)	*P*
		Lag 70-90 days	7	-0.013 (-0.021, -0.005)	0.002	9.50	36.8	0. 147
	Total motility							
		Lag 0-9 days	8	-0.005 (-0.019, 0.008)	0.439	36.95	81.1	<0.001
		Lag 10-14 days	8	-0.010 (-0.022, 0.002)	0.096	42.11	83.4	<0.001
		Lag 70-90 days	8	-0.034 (-0.070, 0.001)	0.060	118.04	94.1	<0.001
	Progressive motility							
		Lag 0-9 days	9	0.005 (-0.005, 0.015)	0. 343	29.65	73.0	<0.001
		Lag 10-14 days	9	-0.006 (-0.014, 0.002)	0.154	23.57	66.1	0. 003
		Lag 70-90 days	9	0.002 (-0.024, 0.029)	0.865	79.36	89.9	<0.001
SO_2	Sperm concentration	Lag 0-9 days	6	-0.071 (-0.494, 0.353)	0.744	46.31	89.2	<0.001
		Lag 10-14 days	6	-0.205 (-0.477, 0.067)	0.139	29.01	82.8	<0.001
		Lag 70-90 days	6	0.418 (-0.292, 1.129)	0.248	85.20	94.1	<0.001
	Total sperm count	Lag 0-9 days	5	-0.105 (-0.348, 0.137)	0.395	8.75	54.3	0. 068
		Lag 10-14 days	5	-0.261 (-0.537, 0.015)	0.063	15.88	74.8	0. 003
		Lag 70-90 days	5	-0.125 (-0.260, 0.010)	0.070	9.31	57.0	0. 054
	Total motility	Lag 0-9 days	4	-0.131 (-0.153, -0.109)	<0.001	0.75	0.0	0. 862
		Lag 10-14 days	4	-0.122 (-0.201, -0.043)	0.002	4.13	27.4	0. 248
		Lag 70-90 days	4	-0.130 (-0.339, 0.079)	0.222	9.67	69.0	0. 022
	Progressive motility	Lag 0-9 days	6	0.130 (-0.088, 0.348)	0.243	59.23	91.6	<0.001
		Lag 10-14 days	6	-0.012 (-0.121, 0.097)	0.826	20.84	76.0	0.001
		Lag 70-90 days	6	0.006 (-0.204, 0.217)	0.955	38.56	87.0	<0.001
NO_2	Sperm concentration	Lag 0-9 days	6	0.001 (-0.035, 0.038)	0.944	12.38	59.6	0.030

Table S5. (*continued*)

Pollution type	Semen parameters	Subgroups	No. of studies	Summary β (95% CI)	*P*	Test of heterogeneity X^2	I^2(%)	*P*
		Lag 10-14 days	6	-0.000 (-0.029, 0.029)	0.989	11.36	56.0	0. 045
		Lag 70-90 days	6	0.074 (-0.076, 0.223)	0.334	74.25	93.3	<0.001
	Total sperm count	Lag 0-9 days	5	-0.006 (-0.031, 0.019)	0.618	5.65	29.2	0. 227
		Lag 10-14 days	5	-0.009 (-0.035, 0.016)	0.484	7.68	47.9	0. 104
		Lag 70-90 days	5	-0.015 (-0.028, -0.002)	0.029	2.90	0.0	0. 575
	Total motility	Lag 0-9 days	4	0.007 (-0.003, 0.017)	0.183	1.41	0.0	0.704
		Lag 10-14 days	4	0.004 (-0.007, 0.016)	0.486	3.54	15.3	0.315
		Lag 70-90 days	4	-0.013 (-0.029, 0.004)	0.128	3.54	15.2	0.316
	Progressive motility	Lag 0-9 days	6	0.043 (0.007, 0.079)	0.019	31.06	83.9	<0.001
		Lag 10-14 days	6	0.008 (-0.014, 0.029)	0.483	14.33	65.1	0. 014
		Lag 70-90 days	6	-0.002 (-0.043, 0.039)	0.918	20.73	75.9	0.001
CO	Sperm concentration	Lag 0-9 days	5	0.002 (-0.006, 0.011)	0.608	16.47	75.7	0.002
		Lag 10-14 days	5	-0.001 (-0.003, 0.001)	0.220	1.73	0..0	0.786
		Lag 70-90 days	5	0.026 (-0.004, 0.056)	0.085	165.14	97.6	<0.001
	Total sperm count	Lag 0-9 days	4	-0.004 (-0.014, 0.005)	0.371	9.50	68.4	0.023
		Lag 10-14 days	4	-0.002 (-0.010, 0.005)	0.573	8.30	63.9	0.040
		Lag 70-90 days	4	-0.008 (-0.023, 0.007)	0.299	18.11	83.4	<0.001
	Total motility	Lag 0-9 days	4	-0.007 (-0.017, 0.003)	0.155	48.90	93.9	<0.001
		Lag 10-14 days	4	-0.007 (-0.015, 0.000)	0.063	44.07	93.2	<0.001
		Lag 70-90 days	4	-0.012 (-0.026, 0.002)	0.105	66.67	95.5	<0.001
	Progressive motility	Lag 0-9 days	5	0.003 (-0.003, 0008)	0.305	34.27	88.3	<0.001
		Lag 10-14 days	5	-0.001 (-0.002, 0.001)	0.532	5.88	32.0	0. 208
		Lag 70-90 days	5	0.001 (-0.005, 0.007)	0.714	19.30	79.3	0.001

Table S5. (*continued*)

Pollution type	Semen parameters	Subgroups	No. of studies	Summary β (95% CI)	*P*	Test of heterogeneity		
						X^2	I^2(%)	*P*
O_3	Sperm concentration	Lag 0-9 days	6	-0.012 (-0.049, 0.024)	0.515	26.09	80.8	<0.001
		Lag 10-14 days	6	-0.021 (-0.047, 0.004)	0.100	20.75	75.9	0. 001
		Lag 70-90 days	6	0.001 (-0.008, 0.009)	0.848	3.41	0.0	0. 637
	Total sperm count	Lag 0-9 days	5	-0.005 (-0.030, 0.020)	0.681	8.52	53.1	0. 074
		Lag 10-14 days	5	-0.013 (-0.034, 0.008)	0.227	10.13	60.5	0. 038
		Lag 70-90 days	5	0.006 (-0.003, 0.015)	0.219	0.74	0.0	0. 947
	Total motility	Lag 0-9 days	4	0.003 (-0.010, 0.017)	0.629	9.29	67.7	0. 026
		Lag 10-14 days	4	0.003 (-0.007, 0.013)	0.529	6.96	56.9	0. 073
		Lag 70-90 days	3	0.008 (-0.004, 0.021)	0.197	0.29	0.0	0. 865
	Progressive motility	Lag 0-9 days	5	-0.003 (-0.015, 0.009)	0.625	11.23	64.4	0.024
		Lag 10-14 days	5	-0.003 (-0.009, 0.003)	0.319	4.35	8.0	0.361
		Lag 70-90 days	5	-0.003 (-0.010, 0.004)	0.352	2.42	0.0	0.660

Abbreviations: $PM_{2.5}$, particulate matter with the diameter ≤2.5 µm; PM_{10}, particulate matter with diameter ≤10 µm; SO_2, sulfur dioxide; NO_2, nitrogen dioxide; CO, carbon monoxide; O_3, ozone; β, correlation coefficient.

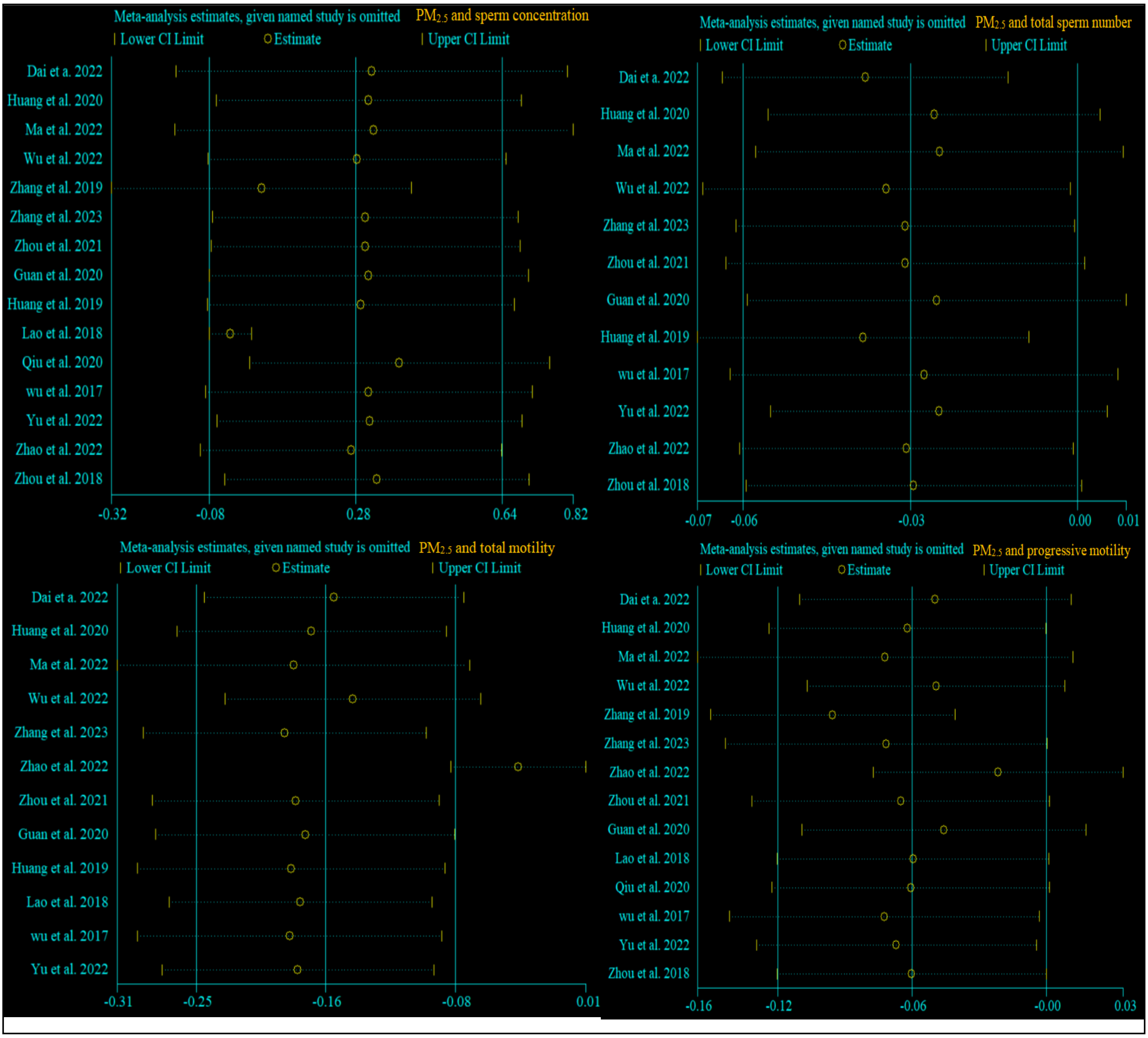

Figure S1a. Sensitivity analyses for the association of $PM_{2.5}$ and semen quality.

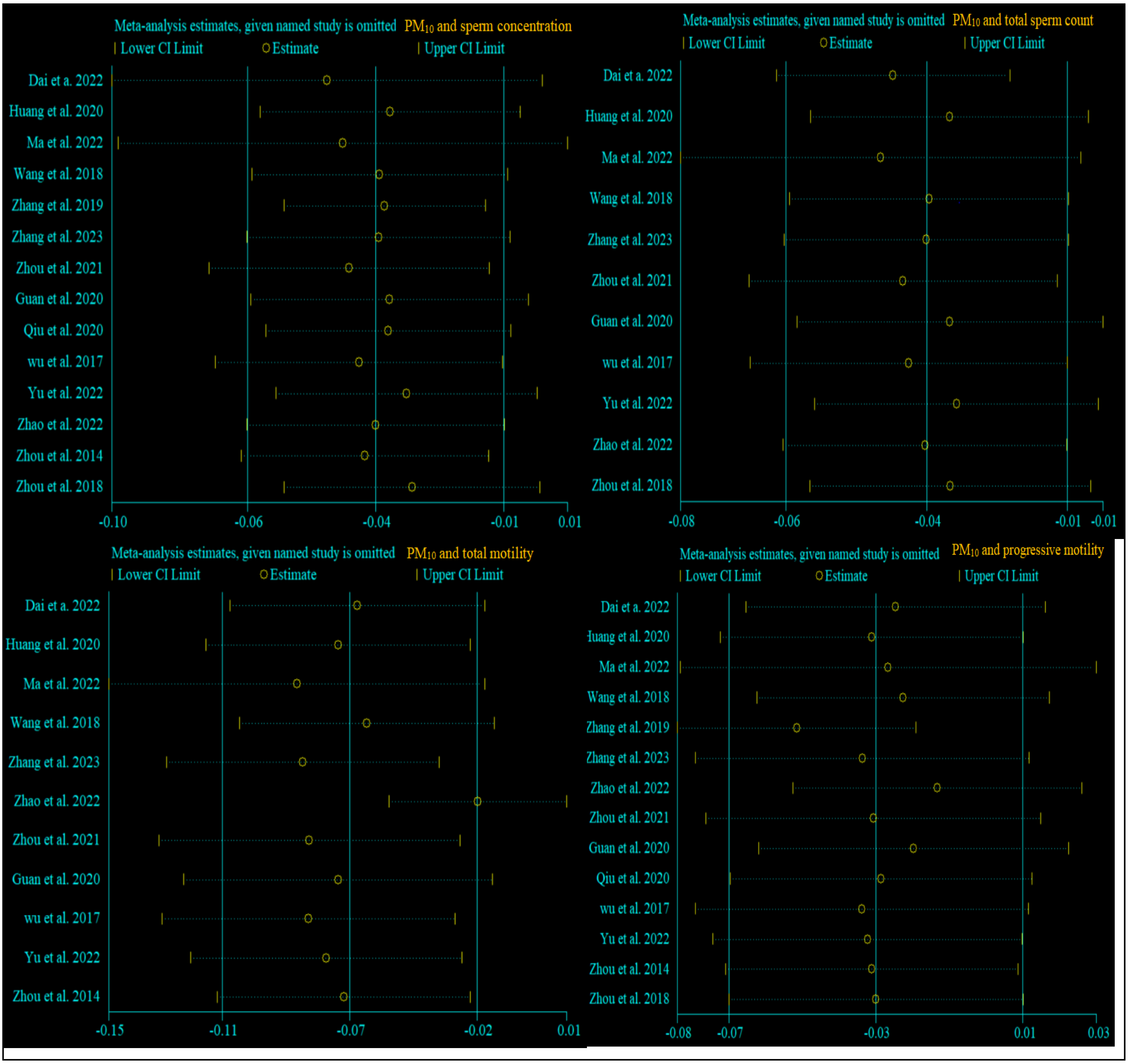

Figure S1b. Sensitivity analyses for the association of PM_{10} and semen quality.

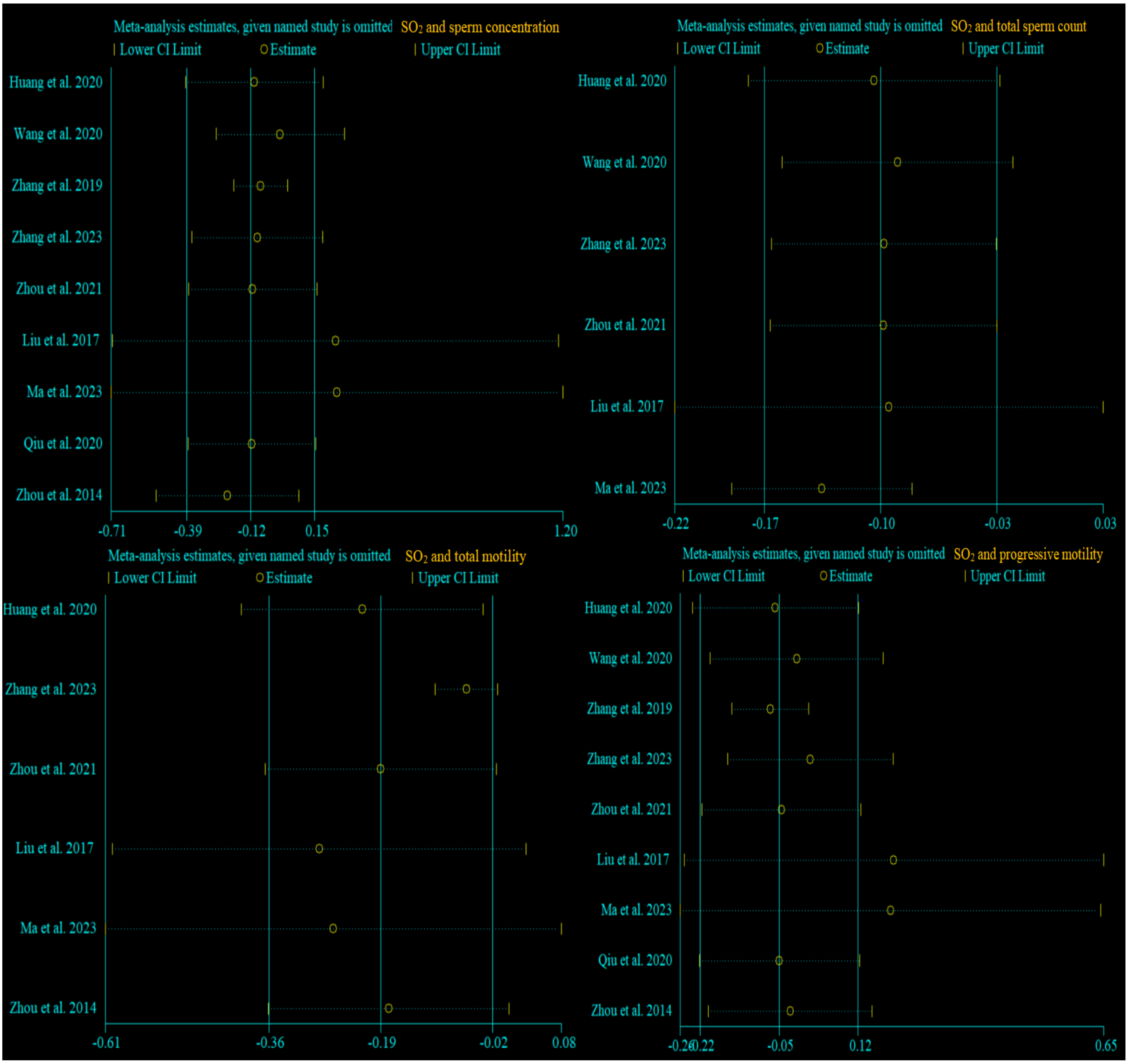

Figure S1c. Sensitivity analyses for the association of SO_2 and semen quality.

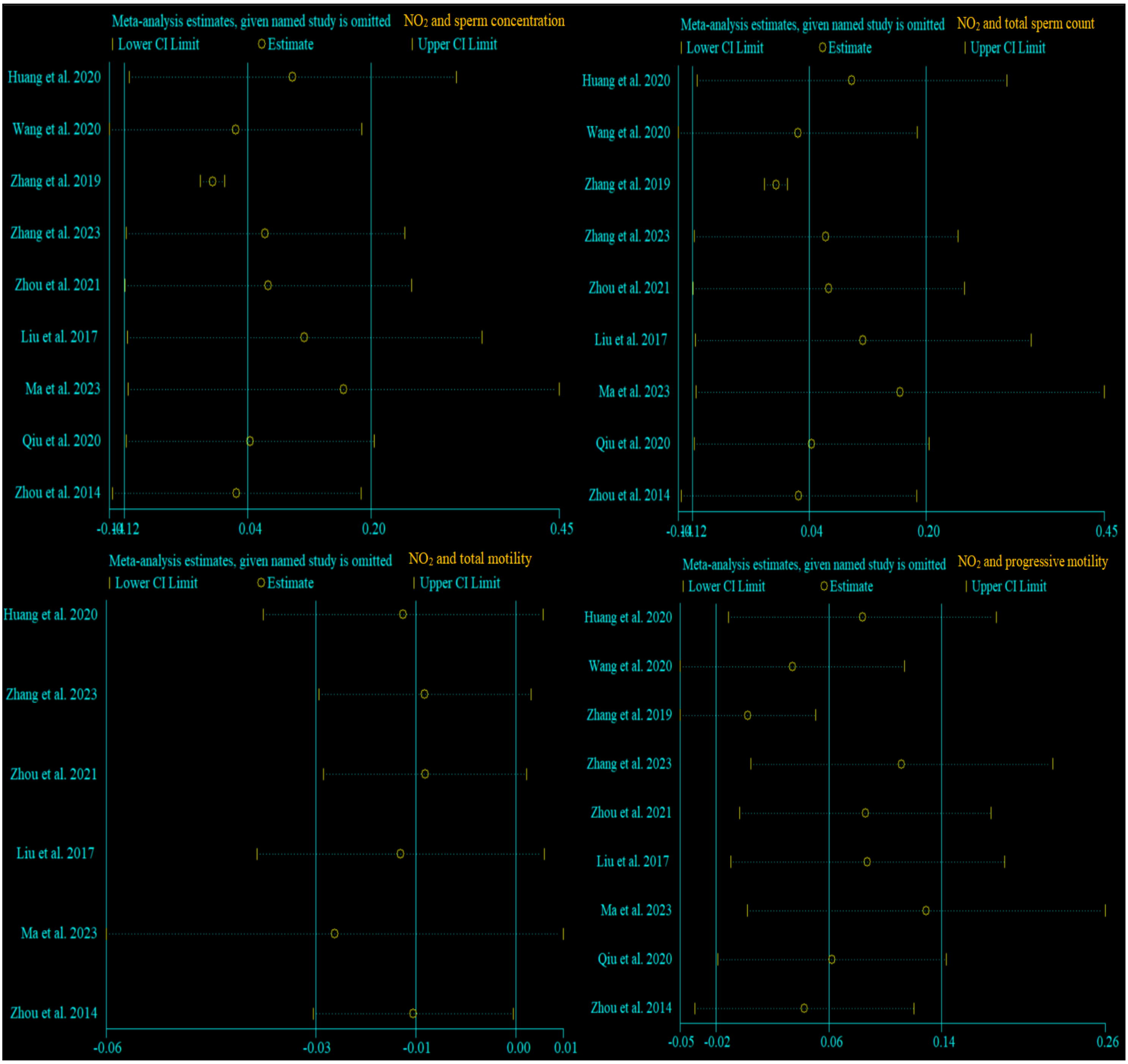

Figure S1d. Sensitivity analyses for the association of NO_2 and semen quality.

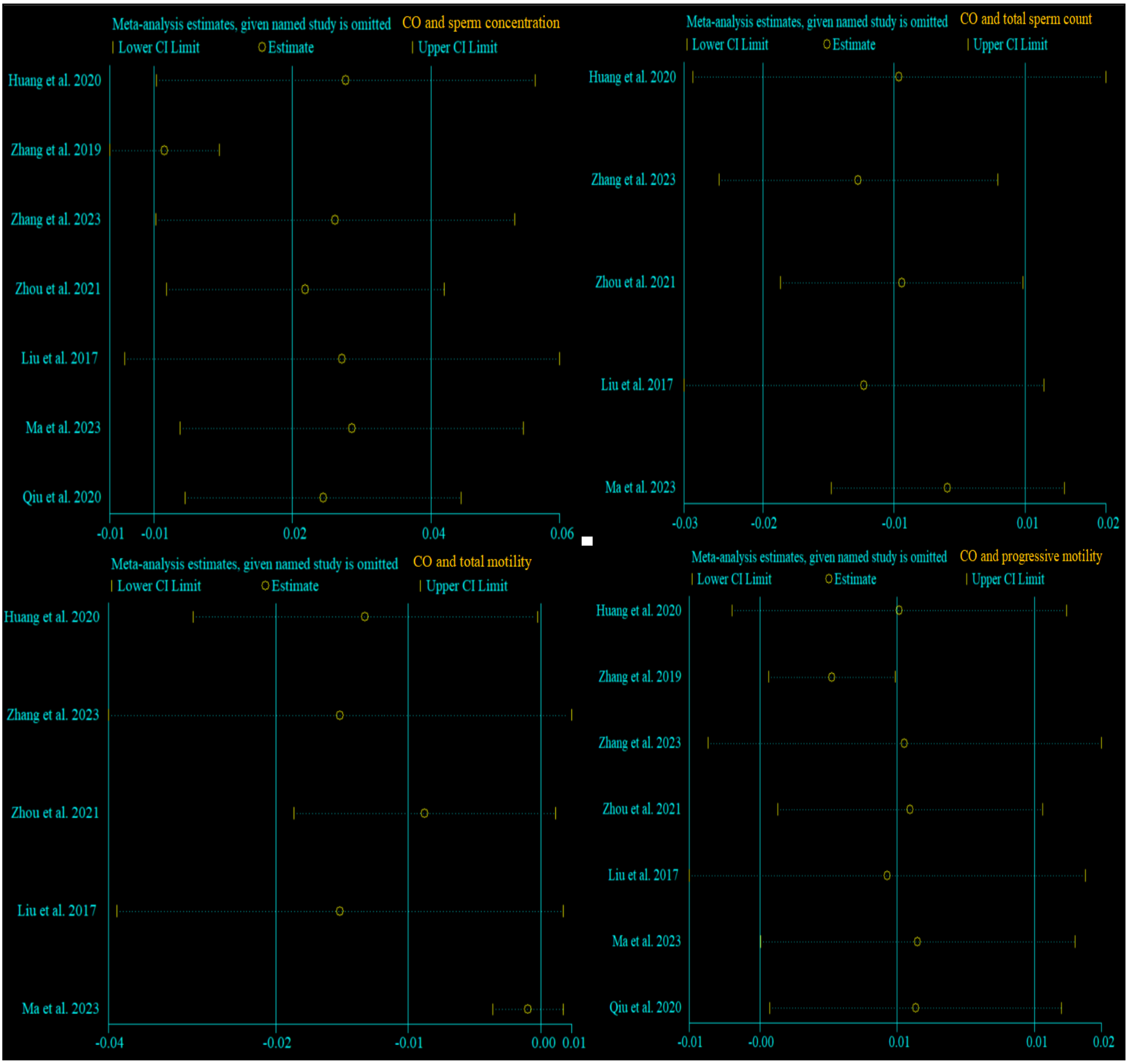

Figure S1e. Sensitivity analyses for the association of CO and semen quality.

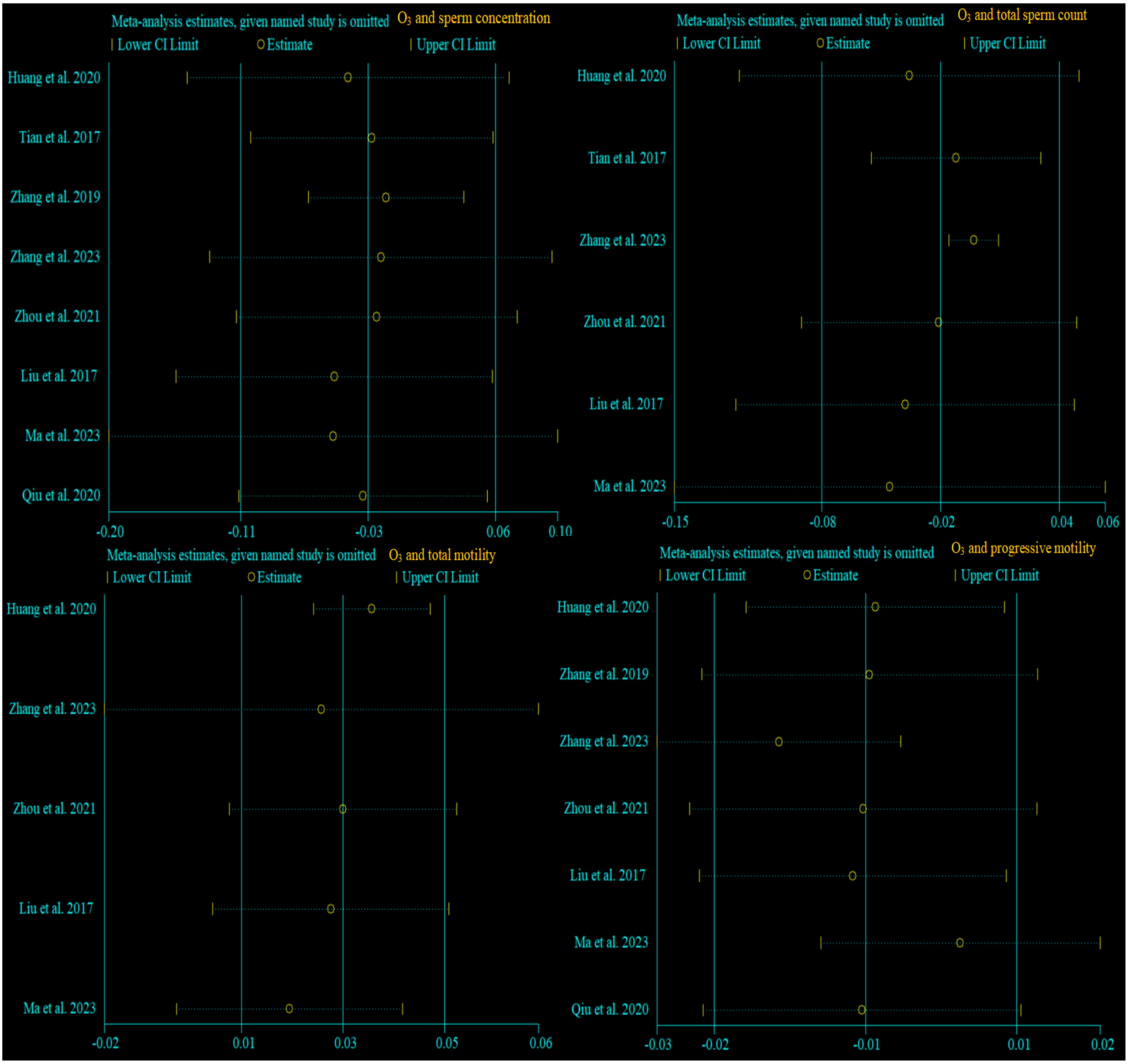

Figure S1f. Sensitivity analyses for the association of O_3 and semen quality.

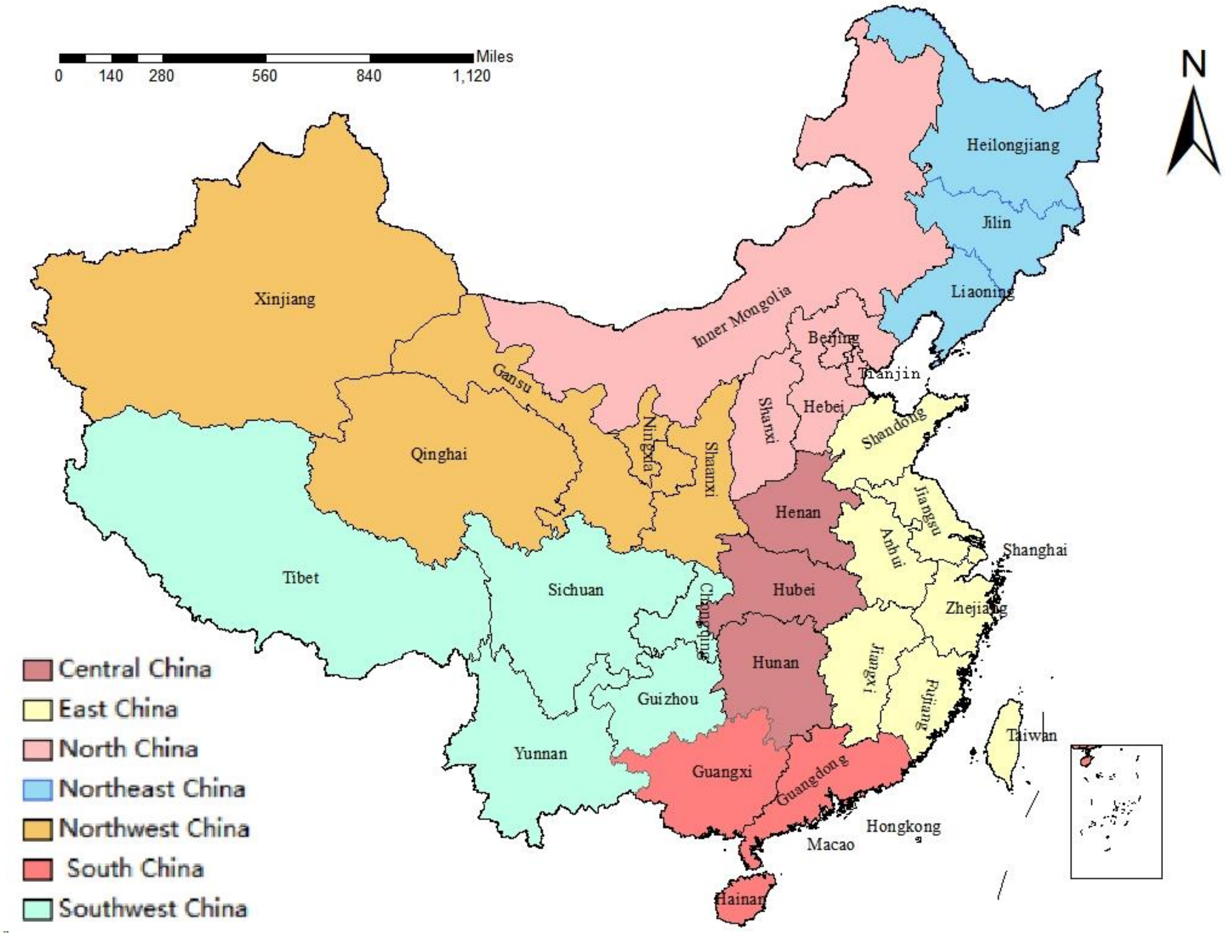

Figure S2. The detailed geographic location of China
